# Supplementary material for: ‘Be on the TEAM’ Study (Teenagers Against Meningitis): protocol for a controlled clinical trial evaluating the impact of 4CMenB or MenB-fHbp vaccination on the pharyngeal carriage of meningococci in adolescents
Source: BMJ Open. 2020 Oct 22;10(10):e037358. doi: 10.1136/bmjopen-2020-037358 (PMC7583083; doi:10.1136/bmjopen-2020-037358)
Supplement: Supplementary data [file bmjopen-2020-037358supp001.pdf]

Be on the TEAM: Teenagers against Meningitis

Evaluating the effect of immunisation with group B meningococcal vaccines on meningococcal carriage

Participant Number: |__| |__| |__| |__| |__|

*****This information is confidential. It will not be seen by school staff or other students *****

Q1. What is your current age in years? 16 ❑ 17 ❑ 18 ❑ 19 ❑

Q2. Are you: male ❑ female ❑ non-binary ❑

Q3. What is your ethnic group?

White ❑
Asian/Asian British ❑
Black/African/Caribbean/Black British ❑
Mixed/multiple ethnic ❑
Other ethnic group ❑

Q4. Do you currently have a cold or sore throat? Yes ❑ No ❑

Q5. Are you currently taking or have you recently stopped taking antibiotics?

Not taken in the past month ❑
Stopped in the last month ❑
Stopped in the last week ❑
Yes currently taking ❑

Q6. How many cigarettes do you smoke in a typical day?

0 ❑ 1-5 ❑ 6-10 ❑ 11-20 ❑ more than 20 ❑

Q7. Does any other person at home smoke cigarettes? No ❑ Yes inside the house ❑ Yes outside the house ❑

Q8. Do you vape (use e-cigarettes)? Yes ❑ No ❑
If YES: How many 10ml refills would you use in a week? <1 ❑ 1-2 ❑ 3-4 ❑ 5-6 ❑ more than 7 ❑
What percentage nicotine do you use most commonly? 0-3 ❑ 4-7 ❑ 8-11 ❑ more than 12 ❑ don't know ❑

Q9. How many days in the last week have you been to a party, pub, bar or nightclub?

0 ❑ 1 ❑ 2-3 ❑ 4-5 ❑ 6-7 ❑

Q10. How many people have you kissed (kissing with tongues, not just lips or cheeks) in the last week?

0 ❑ 1 ❑ 2-3 ❑ 4 or more ❑

Q11. Do you have a regular boyfriend or girlfriend? Yes❑ No❑

If YES: Do they smoke cigarettes? Yes ❑ No ❑ don't know ❑

Or do they vape (use e-cigarettes)? Yes ❑ No ❑ don't know ❑

Q12. Have you ever had the Men ACWY vaccine, also known as Nimenrix or Menveo (when you were 13 or 14 years old)? No ❑ Yes ❑ Unknown ❑

Thank you for completing this questionnaire

Be on the TEAM: Teenagers against Meningitis; REC Ref 18/SC/0055; IRAS 239091; OVG2017/08; Student Questionnaire; Version 3.0 dated --05-Jul-2018
