# Supplementary material for: ‘Be on the TEAM’ Study (Teenagers Against Meningitis): protocol for a controlled clinical trial evaluating the impact of 4CMenB or MenB-fHbp vaccination on the pharyngeal carriage of meningococci in adolescents
Source: BMJ Open. 2020 Oct 22;10(10):e037358. doi: 10.1136/bmjopen-2020-037358 (PMC7583083; doi:10.1136/bmjopen-2020-037358)
Supplement: Author's manuscript [file bmjopen-2020-037358.draft_revisions.pdf]

BMJ Open is committed to open peer review. As part of this commitment we make the peer review history of every article we publish publicly available.

When an article is published we post the peer reviewers' comments and the authors' responses online. We also post the versions of the paper that were used during peer review. These are the versions that the peer review comments apply to.

The versions of the paper that follow are the versions that were submitted during the peer review process. They are not the versions of record or the final published versions. They should not be cited or distributed as the published version of this manuscript.

BMJ Open is an open access journal and the full, final, typeset and author-corrected version of record of the manuscript is available on our site with no access controls, subscription charges or pay-per-view fees (http://bmjopen.bmj.com).

If you have any questions on BMJ Open's open peer review process please email
info.bmjopen@bmj.com

BMJ Open

# The 'Be on the TEAM' Study (Teenagers Against Meningitis): Protocol for a controlled clinical trial evaluating the impact of 4CMenB or MenB-fHbp vaccination on the pharyngeal carriage of meningococci in adolescents.

| | |
|---|---|
| Journal: | *BMJ Open* |
| Manuscript ID | bmjopen-2020-037358 |
| Article Type: | Protocol |
| Date Submitted by the Author: | 12-Feb-2020 |
| Complete List of Authors: | Carr, Jeremy; University of Oxford, Oxford Vaccine Group, Department of Paediatrics<br>Plested, Emma; University of Oxford, Oxford Vaccine Group, Department of Paediatrics; National Institute for Health Oxford Health Biomedical Research Centre<br>Aley, Parvinder; University of Oxford, Oxford Vaccine Group, Department of Paediatrics; National Institute for Health Oxford Health Biomedical Research Centre<br>Camara, Susana; University of Oxford, Oxford Vaccine Group, Department of Paediatrics; National Institute for Health Oxford Health Biomedical Research Centre<br>Davis, Kimberly; University of Oxford, Oxford Vaccine Group, Department of Paediatrics<br>MacLennan, Jenny; University of Oxford, Department of Zoology<br>Gray, Steve; Public Health England, Meningococcal Reference Unit, Manchester Royal Infirmary<br>Faust, Saul; University of Southampton, NIHR Southampton Clinical Research Facility and NIHR Southampton Biomedical Research Centre, University of Southampton Faculty of Medicine and Institute of Life Sciences, University Hospital Southampton NHS Foundation Trust<br>Borrow, Ray; Public Health England, Meningococcal Reference Unit, Manchester Royal Infirmary<br>Christensen, Hannah; University of Bristol, School of Population Health Sciences, Bristol Medical School<br>Trotter, Caroline; University of Cambridge, Department of Veterinary Medicine<br>Maiden, Martin; University of Oxford, Department of Zoology<br>Finn, Adam; University of Bristol, School of Population Health Sciences, Bristol Medical School<br>Snape, Matthew; University of Oxford, Oxford Vaccine Group, Department of Paediatrics; National Institute for Health Oxford Health Biomedical Research Centre |
| Keywords: | INFECTIOUS DISEASES, PUBLIC HEALTH, Paediatric infectious disease & immunisation < PAEDIATRICS, IMMUNOLOGY |
| | |

SCHOLARONE™
Manuscripts

*I, the Submitting Author has the right to grant and does grant on behalf of all authors of the Work (as defined in the below author licence), an exclusive licence and/or a non-exclusive licence for contributions from authors who are: i) UK Crown employees; ii) where BMJ has agreed a CC-BY licence shall apply, and/or iii) in accordance with the terms applicable for US Federal Government officers or employees acting as part of their official duties; on a worldwide, perpetual, irrevocable, royalty-free basis to BMJ Publishing Group Ltd ("BMJ") its licensees and where the relevant Journal is co-owned by BMJ to the co-owners of the Journal, to publish the Work in this journal and any other BMJ products and to exploit all rights, as set out in our licence.*

*The Submitting Author accepts and understands that any supply made under these terms is made by BMJ to the Submitting Author unless you are acting as an employee on behalf of your employer or a postgraduate student of an affiliated institution which is paying any applicable article publishing charge ("APC") for Open Access articles. Where the Submitting Author wishes to make the Work available on an Open Access basis (and intends to pay the relevant APC), the terms of reuse of such Open Access shall be governed by a Creative Commons licence – details of these licences and which Creative Commons licence will apply to this Work are set out in our licence referred to above.*

*Other than as permitted in any relevant BMJ Author's Self Archiving Policies, I confirm this Work has not been accepted for publication elsewhere, is not being considered for publication elsewhere and does not duplicate material already published. I confirm all authors consent to publication of this Work and authorise the granting of this licence.*

For peer review only

# The ‘Be on the TEAM’ Study (Teenagers Against Meningitis): Protocol for a controlled clinical trial evaluating the impact of 4CMenB or MenB-fHbp vaccination on the pharyngeal carriage of meningococci in adolescents.

Jeremy Carr

Corresponding author

Oxford Vaccine Group, Department of Paediatrics, University of Oxford and the National Institute for Health Research (NIHR) Oxford Biomedical Research Centre, Oxford, UK.

Email jeremy.carr@paediatrics.ox.ac.uk

Address Oxford Vaccine Group, Department of Paediatrics, University of Oxford

Centre for Clinical Vaccinology and Tropical Medicine

Churchill Hospital, Churchill Drive

Oxford, Oxfordshire. OX3 7LE

Phone +44 1865 611 400

Emma Plested

Oxford Vaccine Group, Department of Paediatrics, University of Oxford and the National Institute for Health Research (NIHR) Oxford Biomedical Research Centre, Oxford, UK.

emma.plested@paediatrics.ox.ac.uk

Parvinder Aley

Oxford Vaccine Group, Department of Paediatrics, University of Oxford and the National Institute for Health Research (NIHR) Oxford Biomedical Research Centre, Oxford, UK.

parvinder.aley@paediatrics.ox.ac.uk

Susana Camara

Oxford Vaccine Group, Department of Paediatrics, University of Oxford and the National Institute for Health Research (NIHR) Oxford Biomedical Research Centre, Oxford, UK.

susana.camara@paediatrics.ox.ac.uk

Kimberly Davis



For peer review only - http://bmjopen.bmj.com/site/about/guidelines.xhtml

Oxford Vaccine Group, Department of Paediatrics, University of Oxford and the National Institute for Health Research (NIHR) Oxford Biomedical Research Centre, Oxford, UK.

kimberly.davis@paediatrics.ox.ac.uk

Jenny MacLennan

Department of Zoology, University of Oxford, UK.

jenny.maclennan@zoo.ox.ac.uk

Steve Gray

Public Health England Meningococcal Reference Unit, Manchester Royal Infirmary Manchester, UK.

steve.gray@phe.gov.uk

Saul N Faust

NIHR Southampton Clinical Research Facility and NIHR Southampton Biomedical Research Centre, University of Southampton Faculty of Medicine and Institute for Life Sciences, University Hospital Southampton NHS Foundation Trust, Southampton, UK.

s.faust@soton.ac.uk

Ray Borrow

Public Health England Meningococcal Reference Unit, Manchester Royal Infirmary Manchester, UK.

ray.borrow@phe.gov.uk

Hannah Christensen

School of Population Health Sciences, Bristol Medical School, University of Bristol, UK.

hannah.christensen@bristol.ac.uk

Caroline Trotter

Department of Veterinary Medicine, University of Cambridge, UK.

clt56@cam.ac.uk

For peer review only

Martin Maiden

Department of Zoology, University of Oxford, UK.

martin.maiden@zoo.ox.ac.uk

Adam Finn

School of Population Health Sciences, Bristol Medical School, University of Bristol, UK.

adam.finn@bristol.ac.uk

Matthew D Snape

Oxford Vaccine Group, Department of Paediatrics, University of Oxford and the National Institute for Health Research (NIHR) Oxford Biomedical Research Centre, Oxford, UK.

matthew.snape@paediatrics.ox.ac.uk

and the 'Be on the TEAM' investigators (**as collaborators**):

Paul Heath

St George's University Hospital NHS Foundation Trust, UK.

pheath@sgul.ac.uk

David Turner

Centre of Biological Sciences, University of Nottingham & Nottingham Hospitals NHS Trust, UK.

david.turner@nottingham.ac.uk

Elizabeth Whittaker

Imperial College London, UK.

Imperial College Healthcare NHS Trust, London, UK .

e.whittaker@imperial.ac.uk



For peer review only - http://bmjopen.bmj.com/site/about/guidelines.xhtml

Mala Raman

Plymouth Hospitals NHS Trust, UK.

mraman@nhs.net

Stephen Hughes

Manchester University NHS Foundation Trust, UK.

Stephen.Hughes@mft.nhs.uk

Andrew Smith

College of Medical, Veterinary & Life Sciences, University of Glasgow, UK.

Andrew.Smith@glasgow.ac.uk

Claire Cameron

NHS National Services Scotland, Health Protection Scotland, UK.

clairecameron1@nhs.net

David Orr

Lancashire Teaching Hospitals NHS Foundation Trust, UK.

david.orr@lthtr.nhs.uk

David Baxter

Stockport NHS Foundation Trust, UK.

david.baxter@stockport.nhs.uk

Christopher Williams

Health Protection, Public Health Wales, Cardiff, UK.

christopher.williams25@wales.nhs.uk

For peer review only

Rohit Gowda

Maidstone and Tunbridge Wells NHS Trust, UK.

rohit.gowda1@nhs.net

Christos Zipitis

Wrighton, Wigan and Leigh NHS Foundation Trust, UK

christos.zipitis@wwl.nhs.uk

Katy Fidler

Brighton and Sussex Medical School, UK.

Royal Alexandra Children's Hospital, Brighton and Sussex University Hospital NHS Trust, Brighton, UK.

k.fidler@bsms.ac.uk

Sujata Khajuria

Northamptonshire Healthcare NHS Foundation Trust, UK.

sujata.khajuria@nhft.nhs.uk

Manuscript Word Count: 4309

For peer review only

# ABSTRACT

## Introduction

Capsular group B *Neisseria meningitidis* (MenB) is the most common cause of invasive meningococcal disease (IMD) in many parts of the world. A MenB vaccine directed against the polysaccharide capsule remains elusive due to poor immunogenicity and safety concerns. The vaccines licenced for the prevention of MenB disease, 4CMenB (Bexsero®) and MenB-fHbp (Trumenba®), are serogroup B ‘substitute’ vaccines, comprised of subcapsular proteins and are designed to provide protection against most MenB disease-causing strains. In many high-income countries, such as the UK, adolescents are at increased risk of IMD and have the highest rates of meningococcal carriage. Beginning in the late 1990s, immunisation of this age group with the meningococcal group C conjugate vaccine reduced asymptomatic carriage and disrupted transmission of this organism, resulting in lower group C IMD incidence across all age groups. Whether vaccinating teenagers with the novel ‘MenB’ protein-based vaccines will prevent acquisition or reduce duration of carriage and generate herd protection was unknown at the time of vaccine introduction and could not be inferred from the effects of the conjugate vaccines. 4CMenB and MenB-fHbp may also impact on non-MenB disease-causing capsular groups as well as commensal *Neisseria* species. This study is designed to evaluate the impact of vaccination with 4CMenB or MenB-fHbp on oropharyngeal carriage of pathogenic meningococci in teenagers, and consequently the potential for these vaccines to provide broad community protection against MenB disease.

## Methods and Analysis

The ‘Be on the TEAM’ study is a pragmatic, partially-randomised controlled trial of 24000 16-19 year-old students in their penultimate year of secondary school across the UK with regional allocation to a 0+6 month schedule of 4CMenB or MenB-fHBP or to a control group. Culture-confirmed oropharyngeal carriage will be assessed at baseline and at 12 months, following which the control group will be eligible for 4CMenB vaccination. The primary outcome is the carriage prevalence of



For peer review only

potentially pathogenic meningococci (defined as those with genogroups B, C, W, Y, or X), in each vaccine group compared separately to the control group at 12 months post enrolment, i.e. 12 months after the 1st vaccine dose and 6 months after the 2nd vaccine dose. Secondary outcomes include impact on carriage of: genogroup B meningococci; hyperinvasive meningococci; all meningococci; those meningococci expressing vaccine antigens and the impact on carriage of other *Neisseria* species. A sample size of 8000 in each arm will provide 80% power to detect a 30% reduction in meningococcal carriage, assuming genogroup B, C, W, Y, or X meningococci carriage of 3.43%, a design effect of 1.5, a retention rate of 80% and a significance level of 0.05 . Study results will be available in 2021 and will inform UK and international immunisation policy and future vaccine development.

## Ethics and Dissemination

This study is approved by the National Health Service (NHS) South Central Research Ethics Committee (18/SC/0055); the UK Health Research Authority (IRAS ID 239091) and the UK Medicines and Healthcare products Regulatory Agency. Publications arising from this study will be submitted to peer-reviewed journals. Study results will be disseminated in public forums, online, presented at local and international conferences and made available to the participating schools.

## Prospective Clinical Trials Registration

EudraCT: 2017-004609-42

## Keywords
*Neisseria meningitidis*. Meningococcal Infections. Vaccination. 4CMenB. MenB-fHbp.

For peer review

# Article Summary

## Strengths and Limitations of this Study

- The scale of this carriage study will compensate for the known variation in carriage rates that can occur even when consistent methodology is used across multiple sites. The wide geographic distribution of study sites increases applicability to other settings.
- Regional allocation of schools to the same study group may increase the power to detect population effects in discrete social networks.
- The duration of post-immunisation follow up (6 months) and a single post-immunisation sampling point limits the potential to identify a longer-term or more short-lived reduction in carriage, however, the mobility of school-leavers renders longer-term follow up during the highest period of acquisition infeasible in this study.
- This study will inform modelling estimates of potential community-wide impact of adolescent immunisation with either 4CMenB or MenB-fHbp, which are dependent on whether these vaccines induce herd protection.

For peer review only

# Introduction

## Background and Rationale

Capsular group B *Neisseria meningitidis* (MenB) accounts for most cases of invasive meningococcal disease (IMD) in the United Kingdom, Europe, North America, Australia, New Zealand and many countries in Latin America.[1-3] Most IMD cases occur in infants and young children, with a smaller peak in incidence in late adolescence. Despite advances in recognition and management, the global case fatality rate remains 9%, with substantial sequelae in many survivors.[1] Whilst conjugate vaccines that target the polysaccharide capsule of groups A, C, W and Y meningococci are highly effective, a capsule-based vaccine for MenB remains elusive due to poor immunogenicity and potential autoimmunity resulting from homology with surface glycoproteins on human embryonic neural cells.[4] Consequently the vaccines licenced for the prevention of IMD due to MenB, 4CMenB (Bexsero®, GSK) and MenB-fHbp (Trumenba®, Pfizer), target subcapsular proteins. These proteins are highly diverse and have variable density of surface expression. Thus, adequate breadth of coverage relies on a multi-component strategy and potential to generate cross-protective vaccine-induced antibodies. The 4CMenB vaccine contains factor H-binding protein (fHbp) from Sub-family B / Variant 1 fused with the accessory protein GNA2091; *Neisseria* heparin binding antigen (NHBA), Neisserial adhesin A (NadA) fused with the accessory protein GNA 1030; and an outer-membrane vesicle NZ98/254 (PorA 1.4). MenB-fHbp contains two self-lipidated alleles of fHbp, one each from Sub-family A / Variant 3 and Sub-family B / Variant 1. 4CMenB and MenB-fHbp were licensed based on safety and immunogenicity data determined by human serum bactericidal antibody activity (hSBA) as an established correlate of protection.[5,6]

In September 2015, the UK introduced a routine 4CMenB infant immunisation programme, in which all infants born after the 1st of May 2015 are eligible to receive a 2+1 schedule of 4CMenB at 2, 4 and 12 months of age. Ten months after introduction, the effectiveness of the 2 and 4 month doses against



For peer review only - http://bmjopen.bmj.com/site/about/guidelines.xhtml

all MenB IMD was estimated at 83% with very broad uncertainty (95% CI 24.1-95.2), a result consistent with the estimated breadth of coverage from immunogenicity studies.[3] At three years, expected cases of MenB IMD in vaccine eligible children reduced by 75%.[7] Vaccine effectiveness of the 2+1 schedule was 59.1% (95% CI -31.1 – 97.2) against all MenB IMD and 71.2% against vaccine preventable MenB strains. There are no other vaccine effectiveness studies for either 4CMenB or MenB-fHbp.

Herd protection was not observed or expected from the UK MenB infant programme because carriage is very rare in infants as compared to the peak carriage rates that occur in late adolescence and early adulthood.[8] The potential for indirect protection to enhance vaccine impact and cost-effectiveness is exemplified by the meningococcal group C conjugate (MCC) vaccine campaign that included children, adolescents and young adults. A reduction in carriage in this targeted population dramatically reduced IMD caused by serogroup C meningococcus (MenC) in all age groups in the UK.[9] Maintaining this reduction in carriage and therefore MenC IMD in the UK is the primary rationale for an adolescent booster dose of a MenC containing vaccine in early adolescence following infant priming with a single MCC dose given at 12 months of age.

Based on modelling estimates, an infant-only MenB immunisation programme may prevent 26.3% of all-age IMD in the first five years after introduction.[10] If MenB vaccines could reduce meningococcal carriage by 30% and therefore disrupt transmission, a combined infant and adolescent immunisation programme could reduce all-age annual cases by 48.8% at 10 years and 59.7% at 20 years after introduction, enhancing impact and cost-effectiveness.[10] The impact of 4CMenB immunisation on carriage has been studied in two randomised controlled trials (RCT) to date. The 'B Part of It' cluster-randomised controlled trial assessed the impact of immunisation with 4CMenB on carriage in 24269 secondary school students in Australia.[11] There was no impact on carriage of pathogenic genogroups A, B, C, W, Y, or X at 12 months following immunisation. In the UK, 2954 university students were randomised to receive 4CMenB, MenACWY-CRM, or to an active control.[12] This study showed no effect on the primary outcome of carriage prevalence 1 month after a two-dose schedule of 4CMenB.



For peer review only - http://bmjopen.bmj.com/site/about/guidelines.xhtml

Using a composite end point of carriage at any timepoint 3 to 12 months following immunisation, administration of 4CMenB reduced carriage of any meningococcus by 18.2% (95% CI 3.4 - 30.8) and meningococcus groups B, C, W and Y combined by 26.6% (95% CI 10.5 - 39.9). This reduction was primarily due to a fall in carriage of meningococci of capsular groups C, W, and Y, with a non-significant change in capsular group B meningococcal carriage. Only a minority of students at any institution received the vaccine, which reduced the potential impact of herd protection in the cohort. Studies assessing MenB-fHbp carriage impact are limited to observational reports of several small MenB outbreaks in US universities,[13] where a number of interventions including mass-immunisation campaigns halted new cases, however these studies were not specifically designed or powered to show an impact of MenB-fHbp vaccine on carriage.

4CMenB and MenB-fHBP may elicit immune responses against non-MenB meningococci and, for 4CMenB, *Neisseria gonorrhoeae.*[14,15] Vaccine antigens may also be present in commensal *Neisseria* species resident in the oropharynx.[15,16] Whilst cross-protection against meningococci expressing other disease-associated capsular groups is desirable, a broader impact on colonising strains of *N. meningitidis* or potentially protective non-invasive organisms such as *Neisseria lactamica* requires surveillance. Unencapsulated meningococci, which are generally non-pathogenic, may have greater surface exposure of vaccine antigens and therefore be more vulnerable to mucosal clearance than the potentially invasive capsule-expressing meningococci. Natural exposure to these bacteria throughout childhood contributes to the development of immunity against hyper-invasive strains.[17] It is not known whether vaccines that induce immunity to these *Neisseria* species may facilitate an unintended niche for pathogenic meningococci.


For peer review only - http://bmjopen.bmj.com/site/about/guidelines.xhtml

# Methods

## Objectives and Study Design

The 'Be on the TEAM' (Teenagers Against Meningitis) study is a pragmatic, partially-randomised cluster controlled trial that will evaluate the carriage prevalence of pathogenic meningococci in adolescents vaccinated with either 4CMenB or MenB-fHbp, compared with a control group. Participants aged 16 - 19 years will be enrolled in 16 regions across the UK, with each region allocated to: (i) immunisation at 0 and 6 months with 4CMenB ("4CMenB Group"); (ii) immunisation at 0 and 6 months with MenB-fHbp ("MenB-fHbp Group"); or (iii) an unimmunised Control Group who will be eligible for deferred vaccination with 4CMenB. Oropharygneal swab samples (OPSs) will be taken at baseline and at 12 months.

The primary objective is to describe the carriage prevalence of culture-confirmed *N. meningitidis* genogroup B, C, W, Y, X (i.e. those meningococci possessing genes encoding the capsular polysaccharides corresponding to the respective serogroups) at 12 months after baseline vaccination (6 months following the second vaccine dose) in the 4CMenB and MenB-fHbp Groups compared with the unimmunised Control Group. The study will not directly compare the 4CMenB Group with the MenB-fHbp Group.

The secondary objectives are to determine whether immunisation with either 4CMenB or MenB-fHbp affects the carriage prevalence of:

- Genogroup B meningococci;
- Meningococci of other genogroups and capsule null meningococci;
- Strains belonging to hyper-invasive meningococcal lineages;



For peer review only - http://bmjopen.bmj.com/site/about/guidelines.xhtml

- All meningococcal strains;
- Meningococci expressing antigens contained in MenB-fHbp or 4CMenB;
- Other *Neisseria* species.

A further secondary objective will be to determine any difference in rates of new acquisition of carriage between groups as defined in the primary and secondary objectives above.

As a stand-alone exploratory objective, this study will also permit an assessment of the UK adolescent quadrivalent MenACWY vaccination campaign on carriage.[18] This campaign was introduced in 2015 due to a rise in capsular group W meningococcal (MenW) disease. "UKMenCar4", an adolescent carriage study across the same study network in 2014/15 will act as the pre-intervention study. The post-intervention carriage prevalence will be determined by the baseline carriage samples from the Be on the TEAM study.[19]

## Study setting

This is a multi-centre, school-based study in 16 regions across the UK (Figure 1). Students will be aged 16-19 years at time of enrolment, attending school or college Year 12 (in England and Wales) or S5 (in Scotland), or their equivalent. This will include both government (local authority) and independent schools, 6$^{th}$ form colleges, single sex or coeducation schools, boarding or day schools. Study visits will be conducted in schools, although follow-up visits may occur in a community setting if participants have left school.



For peer review only - http://bmjopen.bmj.com/site/about/guidelines.xhtml

## Sample Size

The sample size of 24 000 participants (8000 in each arm) is based on an estimated carriage rate 3.43% for genogroup B, C, W, Y or X in the control group at the 12 month swab. When assuming a retention rate of 80% and a design effect of 1.5, this sample size will provide 80% power to detect a 30% reduction of these genogroups combined, at a significance level of 0.05.

This anticipated carriage rate is based on results from the UKMenCar4 study, which determined carriage prevalence of 1.65% for genogroup B meningococcus, 2.3% for hyper-invasive meningococci and a combined prevalence of 4.2% for genogroup B, C, W, Y, and X meningococci.[19] The figure of 3.43% accounts for a potential 50% reduction in carriage of capsular groups W and Y due to the UK quadrivalent MenACWY vaccination programme; this figure is necessarily an estimate as there are limited data on the impact of the universal adolescent quadrivalent ACWY vaccine campaign on carriage. A previous study in UK university students showed that immunisation with MenACWY-CRM reduced carriage of genogroup C, W or Y meningococci by 27.1% (95% CIs 6.9 - 42.9) and serogroup C, W, or Y by 36.2% (95% CIs 15.6 - 51.7).[12] A further UK university carriage cohort study suggested that the quadrivalent MenACWY vaccine exerts differential effects on capsular groups and did not prevent expansion of meningococci belonging to the hyperinvasive genogroup W clonal complex 11, although this was based on cross sectional surveys rather than a longitudinal design.[20]

## Study Timeline

In order to meet recruitment targets and maximise the interval between immunisation and the final OPS within the structure of the school exam year, the study will recruit across 4 "waves" over 20 months: Wave 1 March-May 2018; Wave 2 September-November 2018; Wave 3 March-May 2019; Wave 4 September-November 2019 (Figure 2). Recruitment will be balanced between study groups


For peer review only - http://bmjopen.bmj.com/site/about/guidelines.xhtml

for each of the ‘Spring’ (March to May) and ‘Autumn’ (September to November) recruiting periods in view of expected seasonal variation in carriage. Completion of the final post-vaccination pharyngeal swab collection is expected by January 2021.

## Eligibility Criteria

Participants will be healthy adolescents aged 16 - 19 years old at time of enrolment, in their penultimate year of participating secondary school or 6$^{th}$ form colleges, who intend to return for a further year of school. Students will be eligible for enrolment if they are willing and able to provide informed consent and meet the further inclusion criteria:

- willing to comply with all study requirements;
- consent for storage of oropharyngeal swabs and any bacterial isolates for future research;
- willing to allow their General Practitioner to be contacted to confirm vaccination status or medical history.

Participants will be ineligible to participate if they have received a course of either 4CMenB or MenB-fHbp in the past (by documentation or self-report). Furthermore, participants in the 4CMenB Group or MenB-fHbp Group will be ineligible if they meet any of the following criteria:

a. History of anaphylaxis to any component of 4CMenB or MenB-fHbp (For 4CMenB only, this includes allergy to latex);
b. Any other significant disease or disorder, which in the opinion of the investigator may: put the participants at risk because of participation in the trial, influence the result of the trial or affect the participant’s ability to participate. Specific examples include haemophilia or medically diagnosed bleeding disorder, or receipt of anticoagulant medication, that prohibits the use of intramuscular injections
c. known or suspected pregnancy.

For peer review only

Participants in the Control Group that fail any of the specific exclusion criteria *a, b* or *c* will be eligible to participate in the assessment of carriage, but will not be able to receive the 4CMenB vaccine after the final OPS. Ineligibility arising during the trial due to criteria *a*, *b* or *c* in the 4CMenB Group or MenB-fHbp Group will allow inclusion for assessment of carriage at the study endpoint but will exclude a participant from vaccination.

# Study Procedures

## Recruitment

Recruitment will occur in participating schools; individual participants will not be directly recruited by the study teams outside of the school setting. Social media and the study website (www.beontheteam.uk ) will be used for study information and awareness, but these platforms will not be used for recruitment. Participants will receive information through a school assembly about meningococcal disease and the study rationale and design. Subsequently, they will receive a written participant information sheet.

## Informed Consent

All participants will be aged 16 years or over, and are therefore self-consenting as per the National Institute of Health Research guidelines.[21] However, parents/guardians will be provided with information about the study via school communication platforms as well as directly by prospective participants. Consent will be taken by clinical staff (registered doctor or nurse) or non-clinical staff who have had appropriate experience and training. Where interested participants have vulnerabilities that may impair their capacity to provide informed consent, additional input or support from the individual's parents/guardians will be sought. If there is ongoing doubt about an individual's ability to provide informed consent, then they will not be enrolled in the study.



For peer review only - http://bmjopen.bmj.com/site/about/guidelines.xhtml

For peer review only

## Randomisation, Allocation and Blinding

This is an open-label partially-randomised study, with group allocation by study site. All schools within the geographical coverage area of each study site will be allocated to the same study arm, both for logistical reasons and because geographic allocation may increase the potential to detect any impact on herd protection. The allocation of initial sites was determined prior to the start of the study (Figure 1). A capacity assessment stratified sites to either the control arm or immunisation sites depending on existing site expertise and the requirements to maintain equal numbers in each study arm. The immunisation sites were randomised to either the MenB-fHbp Group or the 4CMenB Group. Additional sites may be added during the study and will be pragmatically allocated to a study group to ensure balanced recruitment.

## Interventions

All participants will be required to attend three study visits at their school (Figure 3). Following informed consent, a baseline OPS will be taken by study staff using a standardised method of collection. A flocked swab will be used to sweep from one tonsil across the posterior pharynx to the contralateral tonsil. Participants in all groups will complete a brief questionnaire collecting demographic data and risk factors for meningococcal carriage such as smoking and antimicrobial use (see supplementary file).[22] The questionnaire is compatible with previous large-scale UK meningococcal carriage studies conducted in 1999, 2000, 2001, and 2014/15, and the B Part of It Australian study, which will allow an assessment of trends over time.[11,19,23,24] The 4CMenB and MenB-fHbp groups will both receive a two dose vaccine schedule at 0 and 6 months and all groups will have a second OPS taken at 12 months following enrolment. The Control Group will be offered a two dose schedule of 4CMenB vaccination after their second OPS. Individual swab results will not be given to participants, in accordance with previous meningococcal carriage studies, however participants will be contacted confidentially if *Neisseria gonorrhoeae* is detected.



For peer review only - http://bmjopen.bmj.com/site/about/guidelines.xhtml

## Public and Patient Involvement

This study will provide a unique opportunity to engage adolescents on an unprecedented scale to: (i) increase awareness of meningococcal disease; (ii) highlight the importance of immunisation; and, (iii) stimulate an interest in involvement in impact-driven research that potentially benefits the broader community. All Year 12 (or equivalent) students at participating schools will attend assemblies, often involving meningitis survivors and disease prevention advocates. These 'meningitis ambassadors' will discuss their personal experience of meningitis and provide education about signs and symptoms of meningitis. An ethically approved presentation will describe the science behind the study, including information about IMD and the link between vaccination, carriage and herd protection. Communication material used in this study will be developed with input from focus groups run by two meningitis charities, Meningitis Now and the Meningitis Research Foundation, who will also coordinate the provision of 'meningitis ambassadors'. Other initiatives include a social media policy engaging students, schools and research teams with curated social media posts to complement public engagement aims. Combining these strategies will ensure that participants make a fully-informed decision to participate in the study. Furthermore, by involving entire school year cohorts (regardless of enrolment into the study) an estimated 48 000 students across 200 schools will be engaged in a critical conversation about meningitis, research and the benefits of immunisation across the broader community. Study results will be submitted for publication in peer-reviewed journals, and then disseminated directly to the schools involved, as well as on the study social media platforms, and the Meningitis Now and Meningitis Research Foundation websites.



For peer review only - http://bmjopen.bmj.com/site/about/guidelines.xhtml

## Equality and Diversity

The study will be done across 16 regions throughout the UK and will include schools across the range of socioeconomic strata. School types reflect the variety of educational institutions across the UK as detailed in the study setting. Previous diversity information collected as part of UK MenCar4 across the same study network demonstrated that participants were reflective of their communities according to national census data.[25] Inclusion of whole schools, rather than targeting individuals, will encourage equal participation of diverse populations.

For peer review only

## Sample Handling and Laboratory Processes

Collected OPS will be placed immediately into STGG broth (skim milk, tryptone, glucose, glycerine; EO Labs, Bonnybridge, UK), maintained at 2-8$^0$C until frozen at -80$^0$C, within 4 hours of collection (figure 4). Site laboratories will thaw and plate the OPS sample on selective GC-VCAT agar (vancomycin, colistin amphotericin B, trimethoprim; Thermo Fisher Scientific, Basingstoke, UK) at 37$^0$C in 5% $CO_2$ for up to 48 hours. Putative *Neisseria* species (oxidase positive, Gram-negative diplococci) will be subcultured on to Columbia Horse Blood Agar, then stored in a bead-based cryovial (Technical Service Consultants, Lancashire, UK) at -80$^0$C.

The Public Health England Meningococcal Reference Unit (Manchester), will serogroup putative *Neisseria* isolates using an in-house dot-blot ELISA using NIBSC monoclonal antibodies against capsular polysaccharide serogroups B, C, W and Y.[26] DNA will be extracted using a bead-based MagMAX DNA Multi-Sample Ultra Kit (Thermo Fisher Scientific, MA, USA). Total DNA extracts will be normalised prior to whole genome sequencing on the Illumina NovaSeq6000 platform (Oxford Genomic Centre, Wellcome Trust Centre for Human Genetics, University of Oxford).


For peer review only - http://bmjopen.bmj.com/site/about/guidelines.xhtml

Genomes will be assembled using an in-house assembly pipeline (Department of Zoology, University of Oxford) which incorporates Velvet and VelvetOptimiser.[27,28] Assembled draft genome sequences will then be submitted to the open-access online database http://pubMLST.org/neisseria, with isolate providence data and FASTQ files uploaded to the European Nucleotide Archive and the Sequence Read Archive databases. Automated locus annotation using the BIGSdb software platform will be used concurrently with manual curation for classification of isolates including species, clonal complex, lineage, genogroup, and meningococcal typing schemes for multilocus sequence typing (MLST), ribosomal MLST, core genome MLST, and vaccine antigen variants (e.g. Bexsero Antigen Sequence Type, BAST).[29,30] After completion of the study, genomes will be published open access on the pubMLST.org/neisseria database and OPSs, *Neisseria* isolates and all growth on the GC-VCAT media will be stored in an approved facility for use in future research.

## Potential benefits and harms

Participants will benefit from immunisation with a licenced 'MenB' vaccine which they would not otherwise receive in the UK immunisation schedule. Prior to enrolment, participants will receive information about 4CMenB and MenB-fHbp vaccination, according to the licenced product information. As a participant engagement exercise, all participants completing the final swabbing visit will be entered into a prize-draw for headphones. Participants will not receive payments.

## Safety Reporting and Study Oversight

This study involves products used according to their licences. Safety monitoring will focus on detecting any serious adverse events (SAEs) and suspected unexpected serious adverse reactions (SUSARs). Specific SAE exemptions include traumatic injuries; hospital admissions for intoxication, deliberate self-harm or elective management of pre-existing conditions unless in response to a worsening of that



For peer review only - http://bmjopen.bmj.com/site/about/guidelines.xhtml

condition since study enrolment. All SAEs are reported to the Chief Investigator and to the Sponsor, the University of Oxford Clinical Trials and Research Group. The Oxford University Hospitals NHS Foundation Trust / University of Oxford Trials Safety Group will review all new SAEs on a quarterly basis. SUSARs will be reported by the study Chief Investigator to the relevant Competent Authority and to the Research Ethics Committee. The lead site is the Oxford Vaccine Group, Department of Paediatrics, University of Oxford. The Scientific Advisory Board will provide input into study design, statistical analysis and will oversee safety reporting and study conduct in accordance with Good Clinical Practice. The Oxford Vaccine Group on behalf of the Sponsor will act as the study monitor.

## Statistical Methods

A baseline analysis will occur after Wave 2 when the study reaches approximately 50% enrolment. This descriptive analysis of baseline carriage rates will assess the validity of the prospective sample size calculation. There will be no interim analysis of vaccine impact. The principal analysis will be conducted using a modified intention to treat (mITT) approach, including all participants who have had two throat swabs. Participants will be analysed according to allocated group, regardless of vaccine receipt. The design effect was assumed to be 1.5 in the sample size calculations but will be calculated on the actual data collected.

For the primary objective and each of the secondary objectives, carriage rates will be expressed as a mean with two-sided 95% Clopper-Pearson confidence intervals for each group at baseline and at 12 months. The carriage rates in the Men-fHbp and 4CMenB groups will be compared separately to the Control Group. The odds ratio (OR) with two-sided 95% confidence intervals will be calculated using logistic regression via a generalised estimating equations (GEE) model. Model covariates will include age, gender, season of recruitment, school-level quadrivalent MenACWY vaccine uptake and baseline carriage status *a priori*. Additional risk factors found to affect carriage at baseline will also be included. Gender and smoking (smoking, home smoking and vaping) are known to effect both vaccine-induced

For peer review only

immune responses and meningococcal carriage and these interactions will be reported regardless of significance. The level of statistical significance will be 0.05. There will be no imputation of missing data for the principal analysis.

Sensitivity analyses will be done for the primary and each of the secondary objectives and will include: analyses without adjustment or inclusion of covariates for completeness and to confirm the results from the primary analyses; a per-protocol analysis of participants who received both vaccine doses in the 4CMenB and MenB-fHbp groups; adjustment for the month of the outcome measure rather than season of recruitment to further investigate the potential effect of seasonality and; multivariable imputation for missing data. All variables that will be included in the analysis model will be included in the imputation model and will assume data are missing at random.

## Data Collection and Management

Informed consent forms will be completed on paper. Source data entry will be on a standard case report form, either paper based, or direct electronic entry using REDCap, a browser-based electronic data capture record, hosted by the University of Oxford.[33] Participant data are handled in accordance with Good Clinical Practice and the General Data Protection Act. Participants will consent to the storage of de-identified OPSs, isolates and questionnaire data. All identifiable data will be destroyed after the youngest participant in the trial turns 21 years.

# Discussion

This is the first large-scale adolescent meningococcal carriage study to assess the impact of both the 4CMenB and MenB-fHbp vaccines on carriage of pathogenic meningococci as well as commensal

For peer review only

*Neisseria* species. Building on an established meningococcal carriage and vaccine clinical trial network, the backwards compatibility of this study will allow comparisons with four previous carriage studies in 1999, 2000, 2001, and 2014/15. This study will also facilitate an evaluation of the UK adolescent quadrivalent MenACWY vaccination program on carriage since its introduction in 2015. The 16 sites across the UK represent a geographically and demographically diverse population, increasing applicability to broad settings.

The 'B part of It' trial showed that there is no significant difference in carriage of genogroup A, B, C, W, Y and X meningococci between adolescents who received 4CMenB compared with controls.[11] Overall, the carriage of genogroup B meningococcus was low. The 'B Part of It' study has a similar design as a school-based study of 16-18 year olds; however, there are some notable difference between the design of the B Part of It study and the present study, specifically, the 'Be on the TEAM' study:

(i) includes both MenB-fHBP and 4CMenB;

(ii) allocates vaccine arms on a regional basis rather than cluster randomisation at the level of schools, which may increase the potential to observe herd immunity;

(iii) uses a 0 and 6 month vaccine schedule, longer than the 1-2 month interval used in the B Part of It study;

(iv) has a culture-defined endpoint of carriage, compared with direct PCR amplification targeting PorA in the B Part of It Study.

Despite the difference in study designs, cross-study collaborations will enrich understanding of the potential vaccine impact on specific *N. meningitidis* strains and commensal *Neisseria* species by genome level interrogation.



For peer review only - http://bmjopen.bmj.com/site/about/guidelines.xhtml

The short follow-up period of between 6 to 9 months following the second vaccination is a limitation of this study, reducing the ability to observe any potential impact during later periods of high-acquisition risk, e.g. when commencing tertiary education.[31] The large sample size required and mobility of school-leavers limits the feasibility of ongoing carriage surveillance. The secondary endpoint assessing the impact on other *Neisseria* species is based on those present in the oropharynx and culturable on selective media, however all swabs and products of culture will be stored for potential future analysis.

For peer review only

Due for completion in 2021, The 'Be on the TEAM' study will directly inform international MenB immunisation policy. It will contribute to an open-access repository of *Neisseria* spp. genomes and provide a biobank of samples to assist in identifying strategies for future MenB vaccine development.

## Current Study Progress

At the time of writing 24 048 participants have been enrolled from over 120 schools across the UK. Assemblies raising awareness about meningitis, immunisation and research have reached over 40,000 students.

## Acknowledgments

The Scientific Advisory Board: Chair – Sir Brian Greenwood; Deputy Chair – James Stuart; Simon Nadel; Helen Marshall; Helen Bedford; Nick Andrews. Holly Bratcher and Odile Harrison, Department of Zoology, University of Oxford; Melanie Carr, Karen Ford, Hannah Roberts, Yama Farooq, Simon Kerridge and Annabel Giddings, Department of Paediatrics, University of Oxford; Shari Sapuan, St George's University NHS Foundation Trust; Rebecca Ramsay Brighton and Sussex University Hospitals

NHS Foundation Trust; Dr Katrina Cathie, NIHR Southampton Clinical Research Facility and Department of Paediatrics, University Hospital Southampton NHS Foundation Trust. The authors acknowledge Meningitis Now and Meningitis Research Foundation for their input into study branding and design, educational material and organising meningitis ambassadors.

## Author Contributions

The initial study design protocol was developed by MS, AF, MM, CT, HC, RB, SG, JM, PH, SF, KD, SC, PA, EP, JM, JC. CT and HC provided statistical expertise. The manuscript was drafted by JC and MS with input and editing from all authors and collaborators. All authors and collaborators reviewed the final manuscript and approved its contents.

## Funding

This study is independent research funded by the National Institute for Health Research (NIHR) Policy Research Programme ("Be on the TEAM", "Evaluating the effect of 'MenB' vaccination on meningococcal carriage", PR-R18-0117- 21001). Matthew Snape and the Oxford Vaccine Group, Department of Paediatrics, are supported by the NIHR Oxford Biomedical Research Centre. The views expressed in this publication are those of the authors and not necessarily those of the NHS, the NIHR or the Department of Health and Social Care. MenB-fHbp donated by Pfizer. Meningitis Now provided funding for a prize draw to facilitate participant retention. The funders have no role in the study design, conduct, analysis or publications arising from this study.

## Competing Interests

Nothing to disclose: JC; EP; JM; KD; MM; SG. AF reports grants from the Department of Health, during the conduct of the study; grants from Pfizer, grants from GSK, outside the submitted work. HC reports other from Sanofi Pasteur, other from IMS Health, grants from National Institute for Health Research, other from AstraZeneca , grants and other from GSK, outside the submitted work. MS reports grants



For peer review only - http://bmjopen.bmj.com/site/about/guidelines.xhtml

from National Institute for Health Research (NIHR), non-financial support from Pfizer, during the conduct of the study; grants from Pfizer, grants from MCM, grants from GlaxoSmithKline, grants from Novavax, grants from Medimmune, grants from Janssen, outside the submitted work. SC reports salary contribution from the study NIHR grant, during the conduct of the study. SF reports personal fees were paid to his institution (with no personal payment of any kind) from AstraZeneca/Medimmune, Sanofi, Pfizer, Seqirus, Sandoz, Merck, GSK, Alios, Johnson & Johnson, and Merck, outside the submitted work. RB reports grants from University of Oxford via a NIHR grant, during the conduct of the study; contract research on behalf of Public Health England for GSK, Pfizer and Sanofi Pasteur, outside the submitted work.

## Approvals & Clinical Trials Registration

National Health Service (NHS) South Central Research Ethics Committee (18/SC/0055). The UK Health Research Authority (IRAS ID 239091). UK Medicines and Healthcare products Regulatory Agency. Prospective Clinical Trials Registration EudraCT: 2017-004609-42.

## Protocol

Current Protocol Version 4.0 Dated 17-Jun-2019. All protocol modifications will be reported promptly to the NIHR, Department of Health and Human Services, the Scientific Advisory Board and the Principal Investigators. This report was written using the SPIRIT checklist.[32] The study protocol, patient information sheets and an example consent form are publicly available on the study website: www.beontheteam.uk

## Dissemination and Authorship Policy

Publications arising from this study will be submitted to peer-reviewed journals and authorship predetermined according to ICMJE guidelines. Study results will be disseminated in public forums,



For peer review only - http://bmjopen.bmj.com/site/about/guidelines.xhtml

online, presented at local and international conferences and made available to the participating schools.

## Data Sharing Statement

De-identified datasets will be available on reasonable request from the Chief Investigator. Genomes will be made publicly available on the open access http://pubMLST.org/neisseria database following publication of the study results.

## References


1. Sridhar S, Greenwood B, Head C, et al. Global incidence of serogroup B invasive meningococcal disease: a systematic review. *Lancet Infect Dis* 2015;15(11):1334-46. doi: 10.1016/S1473-3099(15)00217-0.
2. Peterson ME, Li Y, Bita A, et al. Meningococcal serogroups and surveillance: a systematic review and survey. *J Glob Health* 2019;9(1):010409. doi: 10.7189/jogh.09.010409.
3. Parikh SR, Andrews NJ, Beebeejaun K, et al. Effectiveness and impact of a reduced infant schedule of 4CMenB vaccine against group B meningococcal disease in England: a national observational cohort study. *The Lancet* 2016;388(10061):2775-82. doi: 10.1016/s0140-6736(16)31921-3.
4. Finne J, Leinonen M, Makela PH. Antigenic similarities between brain components and bacteria causing meningitis. Implications for vaccine development and pathogenesis. *Lancet* 1983;2(8346):355-7.
5. McIntosh ED, Broker M, Wassil J, et al. Serum bactericidal antibody assays - The role of complement in infection and immunity. *Vaccine* 2015;33(36):4414-21. doi: 10.1016/j.vaccine.2015.07.019.
6. Goldschneider I. Human Immunity to the Meningococcus: I. The Role of Humoral Antibodies. *Journal of Experimental Medicine* 1969;129(6):1307-26. doi: 10.1084/jem.129.6.1307.
7. Ladhani SN, Andrews N, Parikh SR, et al. Vaccination of Infants with Meningococcal Group B Vaccine (4CMenB) in England. *N Engl J Med* 2020;382(4):309-17. doi: 10.1056/NEJMoa1901229.
8. Christensen H, May M, Bowen L, et al. Meningococcal carriage by age: a systematic review and meta-analysis. *The Lancet Infectious Diseases* 2010;10(12):853-61. doi: 10.1016/s1473-3099(10)70251-6.
9. Trotter CL, Maiden MC. Meningococcal vaccines and herd immunity: lessons learned from serogroup C conjugate vaccination programs. *Expert Rev Vaccines* 2009;8(7):851-61. doi: 10.1586/erv.09.48.
10. Christensen H, Hickman M, Edmunds WJ, et al. Introducing vaccination against serogroup B meningococcal disease: an economic and mathematical modelling study of potential impact. *Vaccine* 2013;31(23):2638-46. doi: 10.1016/j.vaccine.2013.03.034.
11. Marshall HS, McMillan M, Koehler AP, et al. Meningococcal B Vaccine and Meningococcal Carriage in Adolescents in Australia. *N Engl J Med* 2020;382(4):318-27. doi: 10.1056/NEJMoa1900236.
12. Read RC, Baxter D, Chadwick DR, et al. Effect of a quadrivalent meningococcal ACWY glycoconjugate or a serogroup B meningococcal vaccine on meningococcal carriage: an




For peer review only - http://bmjopen.bmj.com/site/about/guidelines.xhtml

observer-blind, phase 3 randomised clinical trial. *The Lancet* 2014;384(9960):2123-31. doi: 10.1016/s0140-6736(14)60842-4.
13. Soeters HM, Whaley M, Alexander-Scott N, et al. Meningococcal Carriage Evaluation in Response to a Serogroup B Meningococcal Disease Outbreak and Mass Vaccination Campaign at a College-Rhode Island, 2015-2016. *Clin Infect Dis* 2017;64(8):1115-22. doi: 10.1093/cid/cix091.
14. Rodrigues CMC, Lucidarme J, Borrow R, et al. Genomic Surveillance of 4CMenB Vaccine Antigenic Variants among Disease-Causing Neisseria meningitidis Isolates, United Kingdom, 2010-2016. *Emerg Infect Dis* 2018;24(4):673-82. doi: 10.3201/eid2404.171480.
15. Petousis-Harris H, Paynter J, Morgan J, et al. Effectiveness of a group B outer membrane vesicle meningococcal vaccine against gonorrhoea in New Zealand: a retrospective case-control study. *Lancet* 2017;390(10102):1603-10. doi: 10.1016/S0140-6736(17)31449-6.
16. Lucidarme J, Gilchrist S, Newbold LS, et al. Genetic distribution of noncapsular meningococcal group B vaccine antigens in Neisseria lactamica. *Clin Vaccine Immunol* 2013;20(9):1360-9. doi: 10.1128/CVI.00090-13.
17. Pollard AJ, Frasch C. Development of natural immunity to Neisseria meningitidis. *Vaccine* 2001;19(11-12):1327-46.
18. Campbell H, Edelstein M, Andrews N, et al. Emergency Meningococcal ACWY Vaccination Program for Teenagers to Control Group W Meningococcal Disease, England, 2015-2016. *Emerg Infect Dis* 2017;23(7):1184-87. doi: 10.3201/eid2307.170236.
19. Maclennan JM, Maiden MC, Group UMC. UKMENCAR4: A meningococcal carriage study in 21,000 teenagers to understand changing meningococcal epidemiology and evaluate National vaccination policy. International Pathogenic Neisseria Conference; 2016 4th-9th September; Manchester, UK.
20. Oldfield NJ, Green LR, Parkhill J, et al. Limited Impact of Adolescent Meningococcal ACWY Vaccination on Neisseria meningitidis Serogroup W Carriage in University Students. *J Infect Dis* 2018;217(4):608-16. doi: 10.1093/infdis/jix596.
21. Choices NHS. Consent to treatment - Children and young people - NHS Choices: Department of Health; 2016 [updated 2016-28-11. Available from: http://www.nhs.uk/Conditions/Consent-to-treatment/Pages/Children-under-16.aspx.
22. MacLennan J, Kafatos G, Neal K, et al. Social behavior and meningococcal carriage in British teenagers. *Emerg Infect Dis* 2006;12(6):950-7.
23. Maiden MC, Ibarz-Pavon AB, Urwin R, et al. Impact of meningococcal serogroup C conjugate vaccines on carriage and herd immunity. *J Infect Dis* 2008;197(5):737-43. doi: 10.1086/527401.
24. Maiden MC, Stuart JM, Group UKMC. Carriage of serogroup C meningococci 1 year after meningococcal C conjugate polysaccharide vaccination. *Lancet* 2002;359(9320):1829-31. doi: 10.1016/S0140-6736(02)08679-8.
25. Bratcher HB, Rodrigues CMC, Finn A, et al. UKMenCar4: A cross-sectional survey of asymptomatic meningococcal carriage amongst UK adolescents at a period of low invasive meningococcal disease incidence. *Wellcome Open Res* 2019;4:118. doi: 10.12688/wellcomeopenres.15362.2.
26. Public Health England Meningococcal Reference unit User Manual November 2018. Available online https://assets.publishing.service.gov.uk/government/uploads/system/uploads/attachment_data/file/753941/Meningococcal_reference_unit_user_manual.pdf accessed 05-05-2019.
27. Zerbino DR. Using the Velvet de novo assembler for short-read sequencing technologies. *Curr Protoc Bioinformatics* 2010;Chapter 11:Unit 11 5. doi: 10.1002/0471250953.bi1105s31.
28. Zerbino DR, Birney E. Velvet: algorithms for de novo short read assembly using de Bruijn graphs. *Genome Res* 2008;18(5):821-9. doi: 10.1101/gr.074492.107.
29. Jolley KA, Maiden MC. BIGSdb: Scalable analysis of bacterial genome variation at the population level. *BMC Bioinformatics* 2010;11:595. doi: 10.1186/1471-2105-11-595.



For peer review only - http://bmjopen.bmj.com/site/about/guidelines.xhtml

30. Bratcher HB, Corton C, Jolley KA, et al. A gene-by-gene population genomics platform: de novo assembly, annotation and genealogical analysis of 108 representative Neisseria meningitidis genomes. *BMC Genomics* 2014;15:1138. doi: 10.1186/1471-2164-15-1138.
31. Jeppesen CA, Snape MD, Robinson H, et al. Meningococcal carriage in adolescents in the United Kingdom to inform timing of an adolescent vaccination strategy. *J Infect* 2015;71(1):43-52. doi: 10.1016/j.jinf.2015.02.006.
32. Chan AW, Tetzlaff JM, Altman DG, et al. SPIRIT 2013 statement: defining standard protocol items for clinical trials. *Ann Intern Med* 2013;158(3):200-7. doi: 10.7326/0003-4819-158-3-201302050-00583.

For peer review only

**Figure headings and legends**

**Figure 1** Study sites and vaccine group allocation

**Figure 2** Study timeline by recruitment wave and Year 12 / S5 cohort (penultimate year of secondary school) at the time of enrolment. Vaccine groups receive either MenB-fHbp or 4CMenB at 0 + 6 months. See figure 1 for visit window ranges.

**Figure 3** Study design

**Figure 4** Laboratory Analysis Plan. GC-VCAT selective media (vancomycin, colistin, amphotericin B, trimethoprim); MLST multilocus sequence typing; rMLST ribosomal MLST, cgMLST core genome MLST, cc clonal complex

For peer review only

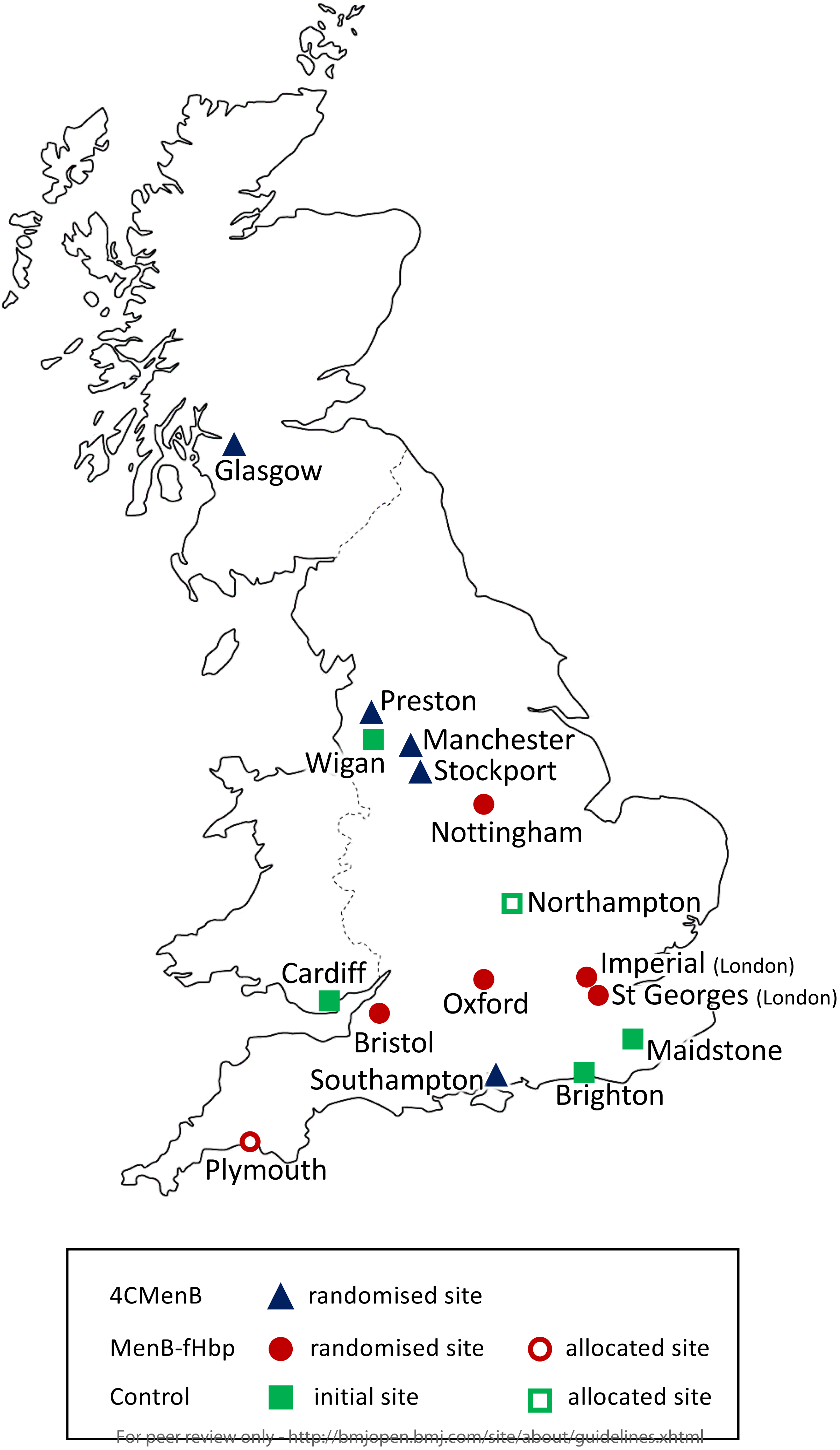
Glasgow
Preston
Manchester
Wigan
Stockport
Nottingham
Northampton
Imperial (London)
St Georges (London)
Cardiff
Oxford
Bristol
Maidstone
Southampton
Brighton
Plymouth
4CMenB
randomised site
MenB-fHbp
randomised site
allocated site
Control
initial site
allocated site

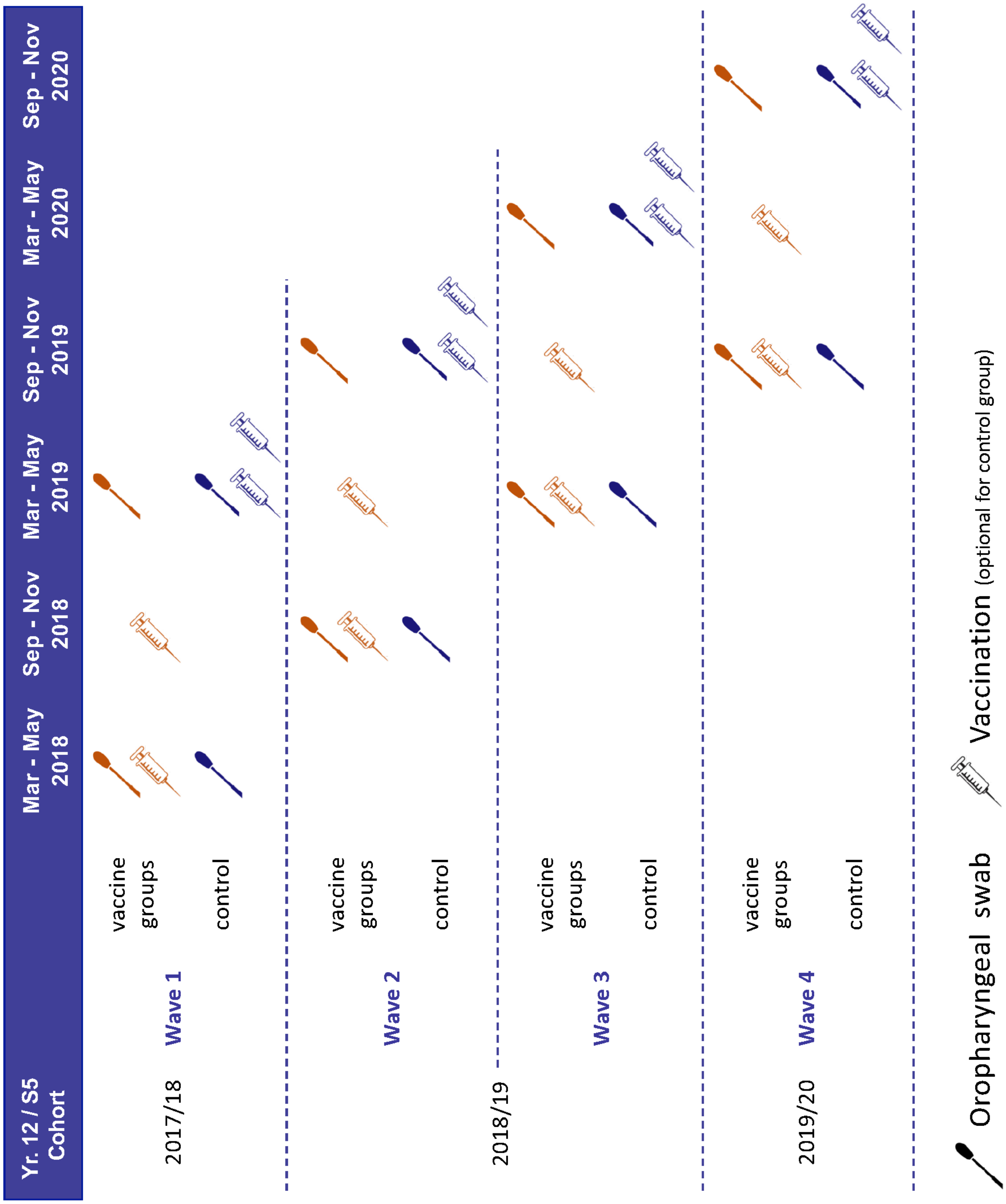
Yr. 12 / S5 Cohort
Mar - May 2018
Sep - Nov 2018
Mar - May 2019
Sep - Nov 2019
Mar - May 2020
Sep - Nov 2020
2017/18
Wave 1
vaccine groups
control
Wave 2
vaccine groups
control
2018/19
Wave 3
vaccine groups
control
2019/20
Wave 4
vaccine groups
control
Oropharyngeal swab
Vaccination (optional for control group)

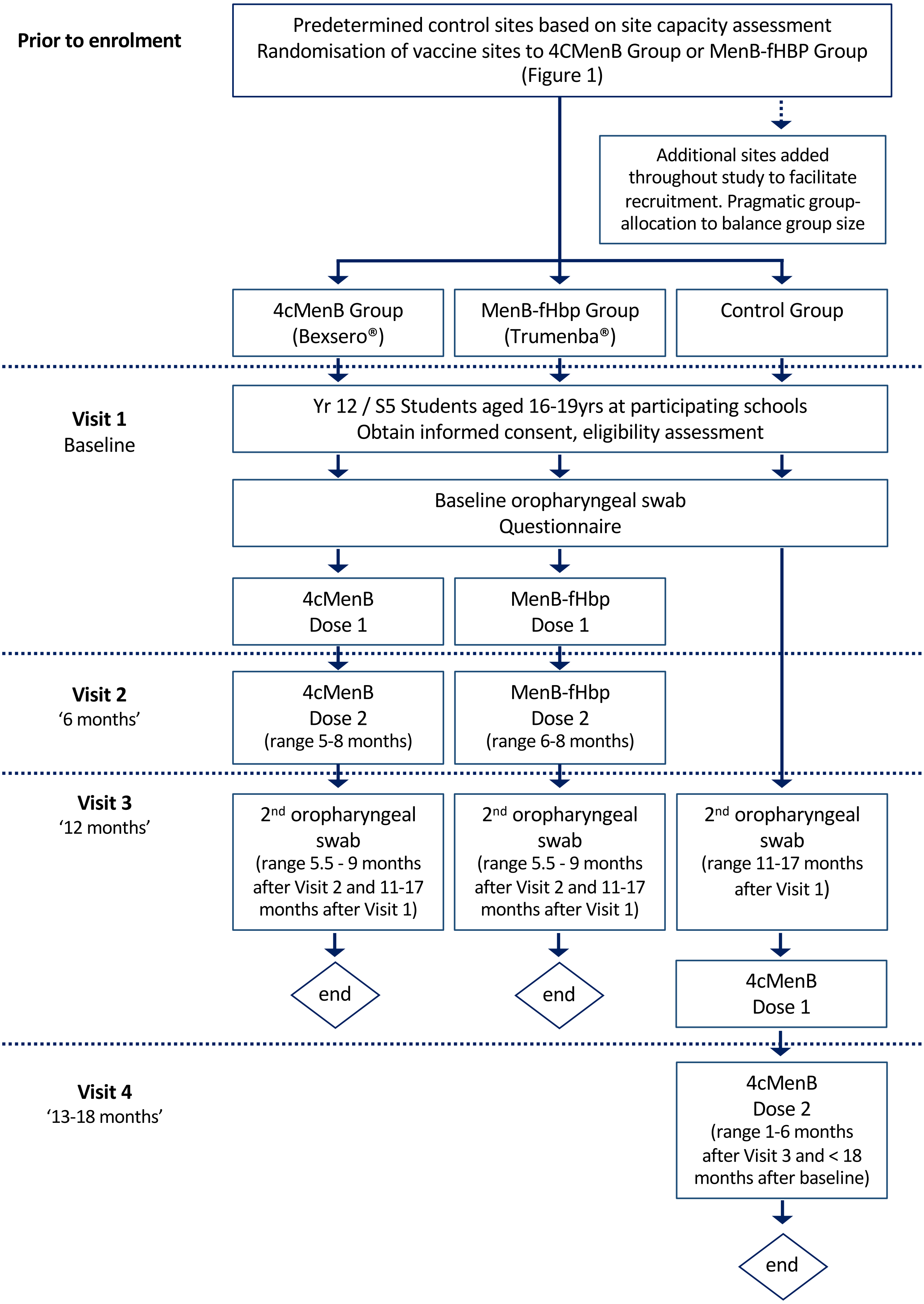
Prior to enrolment
Predetermined control sites based on site capacity assessment
Randomisation of vaccine sites to 4CMenB Group or MenB-fHBP Group (Figure 1)
Additional sites added throughout study to facilitate recruitment. Pragmatic group-allocation to balance group size
4cMenB Group (Bexsero®)
MenB-fHbp Group (Trumenba®)
Control Group
Visit 1
Baseline
Yr 12 / S5 Students aged 16-19yrs at participating schools
Obtain informed consent, eligibility assessment
Baseline oropharyngeal swab
Questionnaire
4cMenB Dose 1
MenB-fHbp Dose 1
Visit 2
'6 months'
4cMenB Dose 2 (range 5-8 months)
MenB-fHbp Dose 2 (range 6-8 months)
Visit 3
'12 months'
2nd oropharyngeal swab (range 5.5 - 9 months after Visit 2 and 11-17 months after Visit 1)
2nd oropharyngeal swab (range 5.5 - 9 months after Visit 2 and 11-17 months after Visit 1)
2nd oropharyngeal swab (range 11-17 months after Visit 1)
end
end
4cMenB Dose 1
Visit 4
'13-18 months'
4cMenB Dose 2 (range 1-6 months after Visit 3 and < 18 months after baseline)
end

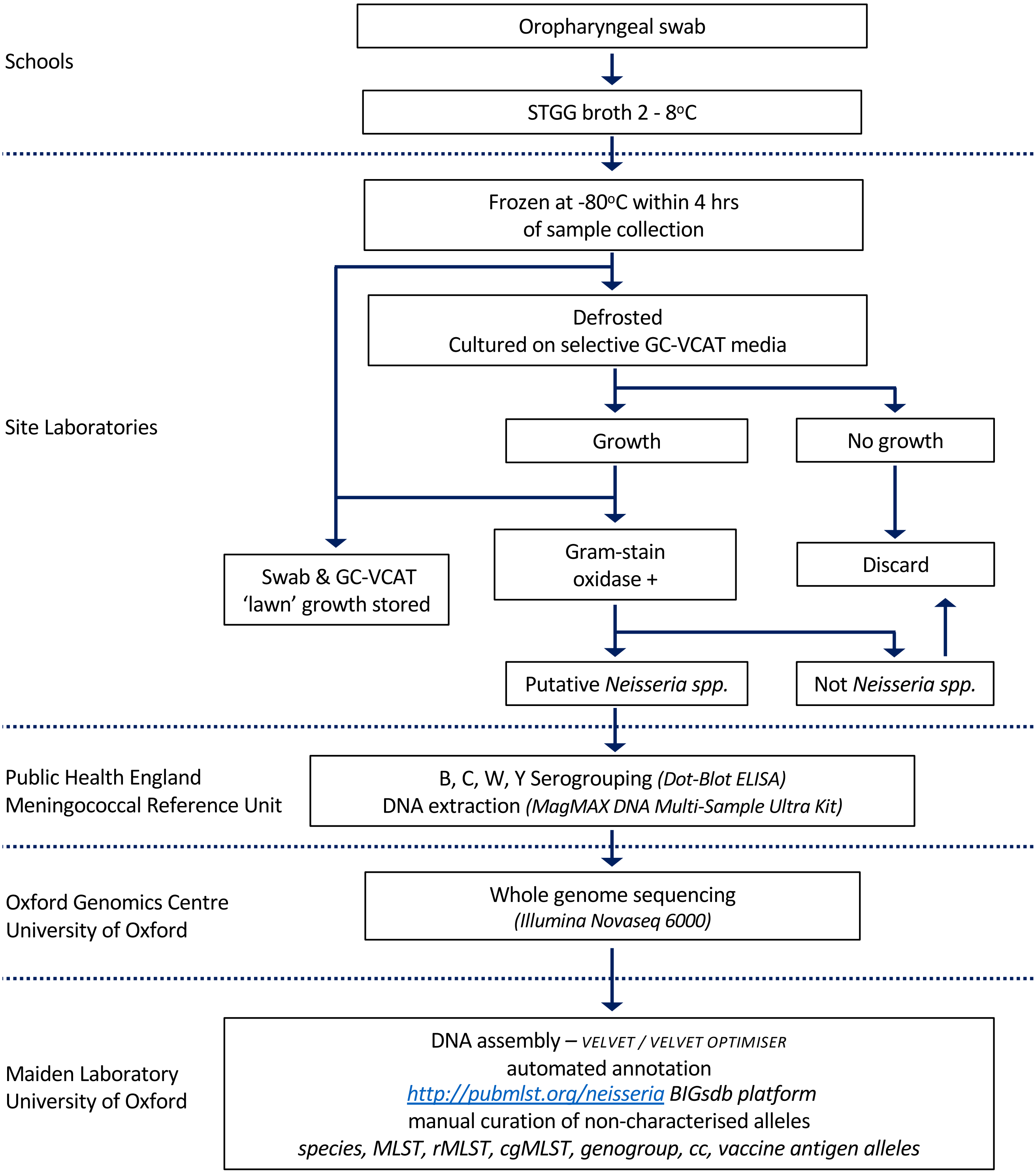
Schools
Oropharyngeal swab
STGG broth 2 - 8ºC
Site Laboratories
Frozen at -80ºC within 4 hrs of sample collection
Defrosted Cultured on selective GC-VCAT media
Growth
No growth
Swab & GC-VCAT 'lawn' growth stored
Gram-stain oxidase +
Discard
Putative Neisseria spp.
Not Neisseria spp.
Public Health England Meningococcal Reference Unit
B, C, W, Y Serogrouping (Dot-Blot ELISA)
DNA extraction (MagMAX DNA Multi-Sample Ultra Kit)
Oxford Genomics Centre University of Oxford
Whole genome sequencing (Illumina Novaseq 6000)
Maiden Laboratory University of Oxford
DNA assembly – VELVET / VELVET OPTIMISER
automated annotation
http://pubmlst.org/neisseria BIGsdb platform
manual curation of non-characterised alleles
species, MLST, rMLST, cgMLST, genogroup, cc, vaccine antigen alleles

# Be on the TEAM: Teenagers against Meningitis

**Evaluating the effect of immunisation with group B meningococcal vaccines on meningococcal carriage**

**Participant Number:** |__| |__| |__| |__| |__|

*****This information is confidential. It will not be seen by school staff or other students *****

**Q1. What is your current age in years?** 16 ❑ 17 ❑ 18 ❑ 19 ❑

**Q2. Are you:** male ❑ female ❑ non-binary ❑

**Q3. What is your ethnic group?**

White ❑
Asian/Asian British ❑
Black/African/Caribbean/Black British ❑
Mixed/multiple ethnic ❑
Other ethnic group ❑

**Q4. Do you currently have a cold or sore throat?** Yes ❑ No ❑

**Q5. Are you currently taking or have you recently stopped taking antibiotics?**

Not taken in the past month ❑
Stopped in the last month ❑
Stopped in the last week ❑
Yes currently taking ❑

**Q6. How many cigarettes do you smoke in a typical day?**

0 ❑ 1-5 ❑ 6-10 ❑ 11-20 ❑ more than 20 ❑

**Q7. Does any other person at home smoke cigarettes?** No ❑ Yes inside the house ❑ Yes outside the house ❑

**Q8. Do you vape (use e-cigarettes)?** Yes ❑ No ❑
**If YES: How many 10ml refills would you use in a week?** <1 ❑ 1-2 ❑ 3-4 ❑ 5-6 ❑ more than 7 ❑
**What percentage nicotine do you use most commonly?** 0-3 ❑ 4-7 ❑ 8-11 ❑ more than 12 ❑ don't know ❑

**Q9. How many days in the last week have you been to a party, pub, bar or nightclub?**

0 ❑ 1 ❑ 2-3 ❑ 4-5 ❑ 6-7 ❑

**Q10. How many people have you kissed (kissing with tongues, not just lips or cheeks) in the last week?**

0 ❑ 1 ❑ 2-3 ❑ 4 or more ❑

**Q11. Do you have a regular boyfriend or girlfriend?** Yes❑ No❑

**If YES: Do they smoke cigarettes?** Yes ❑ No ❑ don't know ❑

**Or do they vape (use e-cigarettes)?** Yes ❑ No ❑ don't know ❑

**Q12. Have you ever had the Men ACWY vaccine, also known as Nimenrix or Menveo (when you were 13 or 14 years old)?** No ❑ Yes ❑ Unknown ❑

**Thank you for completing this questionnaire**

For peer review only - http://bmjopen.bmj.com/site/about/guidelines.xhtml


For peer review only

# Reporting checklist for protocol of a clinical trial.

Based on the SPIRIT guidelines.

## Instructions to authors

Complete this checklist by entering the page numbers from your manuscript where readers will find each of the items listed below.

Your article may not currently address all the items on the checklist. Please modify your text to include the missing information. If you are certain that an item does not apply, please write "n/a" and provide a short explanation.

Upload your completed checklist as an extra file when you submit to a journal.

In your methods section, say that you used the SPIRITreporting guidelines, and cite them as:

Chan A-W, Tetzlaff JM, Altman DG, Laupacis A, Gøtzsche PC, Krleža-Jerić K, Hróbjartsson A, Mann H, Dickersin K, Berlin J, Doré C, Parulekar W, Summerskill W, Groves T, Schulz K, Sox H, Rockhold FW, Rennie D, Moher D. SPIRIT 2013 Statement: Defining standard protocol items for clinical trials. Ann Intern Med. 2013;158(3):200-207

For peer review only

| | | Reporting Item | Page Number |
|---|---|---|---|
| **Administrative information** | | | |
| Title | #1 | Descriptive title identifying the study design, population, interventions, and, if applicable, trial acronym | 1 |
| Trial registration | #2a | Trial identifier and registry name. If not yet registered, name of intended registry | 7 |
| Trial registration: data set | #2b | All items from the World Health Organization Trial Registration Data Set | n/a |
| Protocol version | #3 | Date and version identifier | 25 |
| Funding | #4 | Sources and types of financial, material, and other support | 24 |
| Roles and | #5a | Names, affiliations, and roles of protocol | 24 |

| | | | |
|---|---|---|---|
| responsibilities: contributorship | | contributors | |
| Roles and responsibilities: sponsor contact information | #5b | Name and contact information for the trial sponsor | 20 |
| Roles and responsibilities: sponsor and funder | #5c | Role of study sponsor and funders, if any, in study design; collection, management, analysis, and interpretation of data; writing of the report; and the decision to submit the report for publication, including whether they will have ultimate authority over any of these activities | 24 |
| Roles and responsibilities: committees | #5d | Composition, roles, and responsibilities of the coordinating centre, steering committee, endpoint adjudication committee, data management team, and other individuals or groups overseeing the trial, if applicable (see Item 21a for data monitoring committee) | 20,23 |
| **Introduction** | | | |
| Background and rationale | #6a | Description of research question and justification for undertaking the trial, including summary of relevant studies (published and unpublished) examining benefits and harms for each intervention | 9-12 |
| Background and rationale: choice of comparators | #6b | Explanation for choice of comparators | 12 |
| Objectives | #7 | Specific objectives or hypotheses | 12-13 |
| Trial design | #8 | Description of trial design including type of trial (eg, parallel group, crossover, factorial, single group), allocation ratio, and framework (eg, superiority, equivalence, non-inferiority, exploratory) | 12-13,16 |
| **Methods: Participants,** | | | |

For peer review only

| | | | |
|---|---|---|---|
| **interventions, and outcomes** | | | |
| Study setting | #9 | Description of study settings (eg, community clinic, academic hospital) and list of countries where data will be collected. Reference to where list of study sites can be obtained | 13 |
| Eligibility criteria | #10 | Inclusion and exclusion criteria for participants. If applicable, eligibility criteria for study centres and individuals who will perform the interventions (eg, surgeons, psychotherapists) | 14 |
| Interventions: description | #11a | Interventions for each group with sufficient detail to allow replication, including how and when they will be administered | 17 |
| Interventions: modifications | #11b | Criteria for discontinuing or modifying allocated interventions for a given trial participant (eg, drug dose change in response to harms, participant request, or improving / worsening disease) | 17, 20 |
| Interventions: adherance | #11c | Strategies to improve adherence to intervention protocols, and any procedures for monitoring adherence (eg, drug tablet return; laboratory tests) | 19 |
| Interventions: concomitant care | #11d | Relevant concomitant care and interventions that are permitted or prohibited during the trial | n/a |
| Outcomes | #12 | Primary, secondary, and other outcomes, including the specific measurement variable (eg, systolic blood pressure), analysis metric (eg, change from baseline, final value, time to event), method of aggregation (eg, median, proportion), and time point for each outcome. Explanation of the clinical relevance of chosen efficacy and harm outcomes is strongly recommended | 12, 13 |
| Participant timeline | #13 | Time schedule of enrolment, interventions (including any run-ins and washouts), assessments, and visits for participants. A schematic diagram is highly recommended (see Figure) | 14 |

For peer review only

| | | | |
|---|---|---|---|
| Sample size | #14 | Estimated number of participants needed to achieve study objectives and how it was determined, including clinical and statistical assumptions supporting any sample size calculations | 13 |
| Recruitment | #15 | Strategies for achieving adequate participant enrolment to reach target sample size | 13,14 |
| **Methods: Assignment of interventions (for controlled trials)** | | | |
| Allocation: sequence generation | #16a | Method of generating the allocation sequence (eg, computer-generated random numbers), and list of any factors for stratification. To reduce predictability of a random sequence, details of any planned restriction (eg, blocking) should be provided in a separate document that is unavailable to those who enrol participants or assign interventions | 16 |
| Allocation concealment mechanism | #16b | Mechanism of implementing the allocation sequence (eg, central telephone; sequentially numbered, opaque, sealed envelopes), describing any steps to conceal the sequence until interventions are assigned | n/a |
| Allocation: implementation | #16c | Who will generate the allocation sequence, who will enrol participants, and who will assign participants to interventions | 16 |
| Blinding (masking) | #17a | Who will be blinded after assignment to interventions (eg, trial participants, care providers, outcome assessors, data analysts), and how | n/a |
| Blinding (masking): emergency unblinding | #17b | If blinded, circumstances under which unblinding is permissible, and procedure for revealing a participant's allocated intervention during the trial | n/a |
| **Methods: Data collection,** | | | |

For peer review only

| | | | |
|---|---|---|---|
| **management, and analysis** | | | |
| Data collection plan | #18a | Plans for assessment and collection of outcome, baseline, and other trial data, including any related processes to promote data quality (eg, duplicate measurements, training of assessors) and a description of study instruments (eg, questionnaires, laboratory tests) along with their reliability and validity, if known. Reference to where data collection forms can be found, if not in the protocol | 16-17, 21 |
| Data collection plan: retention | #18b | Plans to promote participant retention and complete follow-up, including list of any outcome data to be collected for participants who discontinue or deviate from intervention protocols | 21 |
| Data management | #19 | Plans for data entry, coding, security, and storage, including any related processes to promote data quality (eg, double data entry; range checks for data values). Reference to where details of data management procedures can be found, if not in the protocol | 21 |
| Statistics: outcomes | #20a | Statistical methods for analysing primary and secondary outcomes. Reference to where other details of the statistical analysis plan can be found, if not in the protocol | 20-21 |
| Statistics: additional analyses | #20b | Methods for any additional analyses (eg, subgroup and adjusted analyses) | 20-21 |
| Statistics: analysis population and missing data | #20c | Definition of analysis population relating to protocol non-adherence (eg, as randomised analysis), and any statistical methods to handle missing data (eg, multiple imputation) | 20-21 |
| **Methods: Monitoring** | | | |
| Data monitoring: formal committee | #21a | Composition of data monitoring committee (DMC); summary of its role and reporting structure; | 20. |

For peer review only

| | | | |
|---|---|---|---|
| | | statement of whether it is independent from the sponsor and competing interests; and reference to where further details about its charter can be found, if not in the protocol. Alternatively, an explanation of why a DMC is not needed | |
| Data monitoring: interim analysis | #21b | Description of any interim analyses and stopping guidelines, including who will have access to these interim results and make the final decision to terminate the trial | 20-21 |
| Harms | #22 | Plans for collecting, assessing, reporting, and managing solicited and spontaneously reported adverse events and other unintended effects of trial interventions or trial conduct | 20 |
| Auditing | #23 | Frequency and procedures for auditing trial conduct, if any, and whether the process will be independent from investigators and the sponsor | 20 & full protocol |
| **Ethics and dissemination** | | | |
| Research ethics approval | #24 | Plans for seeking research ethics committee / institutional review board (REC / IRB) approval | 25 |
| Protocol amendments | #25 | Plans for communicating important protocol modifications (eg, changes to eligibility criteria, outcomes, analyses) to relevant parties (eg, investigators, REC / IRBs, trial participants, trial registries, journals, regulators) | 25 |
| Consent or assent | #26a | Who will obtain informed consent or assent from potential trial participants or authorised surrogates, and how (see Item 32) | 15 & 16 |
| Consent or assent: ancillary studies | #26b | Additional consent provisions for collection and use of participant data and biological specimens in ancillary studies, if applicable | 15 & 16 |
| Confidentiality | #27 | How personal information about potential and enrolled participants will be collected, shared, and | 21 |

For peer review only

| | | | |
|---|---|---|---|
| | | maintained in order to protect confidentiality before, during, and after the trial | |
| Declaration of interests | #28 | Financial and other competing interests for principal investigators for the overall trial and each study site | 25 |
| Data access | #29 | Statement of who will have access to the final trial dataset, and disclosure of contractual agreements that limit such access for investigators | 26 |
| Ancillary and post trial care | #30 | Provisions, if any, for ancillary and post-trial care, and for compensation to those who suffer harm from trial participation | n/a detailed in full protocol & patient information sheet |
| Dissemination policy: trial results | #31a | Plans for investigators and sponsor to communicate trial results to participants, healthcare professionals, the public, and other relevant groups (eg, via publication, reporting in results databases, or other data sharing arrangements), including any publication restrictions | 26 |
| Dissemination policy: authorship | #31b | Authorship eligibility guidelines and any intended use of professional writers | 26 |
| Dissemination policy: reproducible research | #31c | Plans, if any, for granting public access to the full protocol, participant-level dataset, and statistical code | 26 |
| **Appendices** | | | |
| Informed consent materials | #32 | Model consent form and other related documentation given to participants and authorised surrogates | 26 & study website |
| Biological specimens | #33 | Plans for collection, laboratory evaluation, and storage of biological specimens for genetic or molecular analysis in the current trial and for future use in ancillary studies, if applicable | 18-19 |

Notes:

For peer review only

- 30: n/a detailed in full protocol & patient information sheet
- 32: 26 & study website The SPIRIT checklist is distributed under the terms of the Creative Commons Attribution License CC-BY-ND 3.0. This checklist was completed on 30. January 2020 using https://www.goodreports.org/, a tool made by the EQUATOR Network in collaboration with Penelope.ai

For peer review only

BMJ Open

# The 'Be on the TEAM' Study (Teenagers Against Meningitis): Protocol for a controlled clinical trial evaluating the impact of 4CMenB or MenB-fHbp vaccination on the pharyngeal carriage of meningococci in adolescents.

| | |
|---|---|
| Journal: | *BMJ Open* |
| Manuscript ID | bmjopen-2020-037358.R1 |
| Article Type: | Protocol |
| Date Submitted by the Author: | 10-Jul-2020 |
| Complete List of Authors: | Carr, Jeremy; University of Oxford, Oxford Vaccine Group, Department of Paediatrics<br>Plested, Emma; University of Oxford, Oxford Vaccine Group, Department of Paediatrics; National Institute for Health Oxford Health Biomedical Research Centre<br>Aley, Parvinder; University of Oxford, Oxford Vaccine Group, Department of Paediatrics; National Institute for Health Oxford Health Biomedical Research Centre<br>Camara, Susana; University of Oxford, Oxford Vaccine Group, Department of Paediatrics; National Institute for Health Oxford Health Biomedical Research Centre<br>Davis, Kimberly; University of Oxford, Oxford Vaccine Group, Department of Paediatrics<br>MacLennan, Jenny; University of Oxford, Department of Zoology<br>Gray, Steve; Public Health England, Meningococcal Reference Unit, Manchester Royal Infirmary<br>Faust, Saul; University of Southampton, NIHR Southampton Clinical Research Facility and NIHR Southampton Biomedical Research Centre, University of Southampton Faculty of Medicine and Institute of Life Sciences, University Hospital Southampton NHS Foundation Trust<br>Borrow, Ray; Public Health England, Meningococcal Reference Unit, Manchester Royal Infirmary<br>Christensen, Hannah; University of Bristol, School of Population Health Sciences, Bristol Medical School<br>Trotter, Caroline; University of Cambridge, Department of Veterinary Medicine<br>Maiden, Martin; University of Oxford, Department of Zoology<br>Finn, Adam; University of Bristol, School of Population Health Sciences, Bristol Medical School<br>Snape, Matthew; University of Oxford, Oxford Vaccine Group, Department of Paediatrics; National Institute for Health Oxford Health Biomedical Research Centre |
| <b>Primary Subject Heading</b>: | Public health |
| Secondary Subject Heading: | Infectious diseases, Immunology (including allergy), Genetics and genomics, Epidemiology |

| Keywords: | INFECTIOUS DISEASES, PUBLIC HEALTH, Paediatric infectious disease & immunisation < PAEDIATRICS, IMMUNOLOGY |
| --- | --- |
| | |

For peer review only - http://bmjopen.bmj.com/site/about/guidelines.xhtml

*I, the Submitting Author has the right to grant and does grant on behalf of all authors of the Work (as defined in the below author licence), an exclusive licence and/or a non-exclusive licence for contributions from authors who are: i) UK Crown employees; ii) where BMJ has agreed a CC-BY licence shall apply, and/or iii) in accordance with the terms applicable for US Federal Government officers or employees acting as part of their official duties; on a worldwide, perpetual, irrevocable, royalty-free basis to BMJ Publishing Group Ltd ("BMJ") its licensees and where the relevant Journal is co-owned by BMJ to the co-owners of the Journal, to publish the Work in this journal and any other BMJ products and to exploit all rights, as set out in our licence.*

*The Submitting Author accepts and understands that any supply made under these terms is made by BMJ to the Submitting Author unless you are acting as an employee on behalf of your employer or a postgraduate student of an affiliated institution which is paying any applicable article publishing charge ("APC") for Open Access articles. Where the Submitting Author wishes to make the Work available on an Open Access basis (and intends to pay the relevant APC), the terms of reuse of such Open Access shall be governed by a Creative Commons licence – details of these licences and which Creative Commons licence will apply to this Work are set out in our licence referred to above.*

*Other than as permitted in any relevant BMJ Author's Self Archiving Policies, I confirm this Work has not been accepted for publication elsewhere, is not being considered for publication elsewhere and does not duplicate material already published. I confirm all authors consent to publication of this Work and authorise the granting of this licence.*

For peer review only

# The 'Be on the TEAM' Study (Teenagers Against Meningitis): Protocol for a controlled clinical trial evaluating the impact of 4CMenB or MenB-fHbp vaccination on the pharyngeal carriage of meningococci in adolescents.


Jeremy Carr

Corresponding author

Oxford Vaccine Group, Department of Paediatrics, University of Oxford and the National Institute for Health Research (NIHR) Oxford Biomedical Research Centre, Oxford, UK.

Email jeremy.carr@paediatrics.ox.ac.uk

Address Oxford Vaccine Group, Department of Paediatrics, University of Oxford

Centre for Clinical Vaccinology and Tropical Medicine

Churchill Hospital, Churchill Drive

Oxford, Oxfordshire. OX3 7LE

Phone +44 1865 611 400

Emma Plested

Oxford Vaccine Group, Department of Paediatrics, University of Oxford and the National Institute for Health Research (NIHR) Oxford Biomedical Research Centre, Oxford, UK.

emma.plested@paediatrics.ox.ac.uk

Parvinder Aley

Oxford Vaccine Group, Department of Paediatrics, University of Oxford and the National Institute for Health Research (NIHR) Oxford Biomedical Research Centre, Oxford, UK.

parvinder.aley@paediatrics.ox.ac.uk

Susana Camara

Oxford Vaccine Group, Department of Paediatrics, University of Oxford and the National Institute for Health Research (NIHR) Oxford Biomedical Research Centre, Oxford, UK.

susana.camara@paediatrics.ox.ac.uk

Kimberly Davis




For peer review only - http://bmjopen.bmj.com/site/about/guidelines.xhtml

Oxford Vaccine Group, Department of Paediatrics, University of Oxford and the National Institute for Health Research (NIHR) Oxford Biomedical Research Centre, Oxford, UK.

kimberly.davis@paediatrics.ox.ac.uk

Jenny MacLennan

Department of Zoology, University of Oxford, UK.

jenny.maclennan@zoo.ox.ac.uk

Steve Gray

Public Health England Meningococcal Reference Unit, Manchester Royal Infirmary Manchester, UK.

steve.gray@phe.gov.uk

Saul N Faust

NIHR Southampton Clinical Research Facility and NIHR Southampton Biomedical Research Centre, University of Southampton Faculty of Medicine and Institute for Life Sciences, University Hospital Southampton NHS Foundation Trust, Southampton, UK.

s.faust@soton.ac.uk

Ray Borrow

Public Health England Meningococcal Reference Unit, Manchester Royal Infirmary Manchester, UK.

ray.borrow@phe.gov.uk

Hannah Christensen

School of Population Health Sciences, Bristol Medical School, University of Bristol, UK.

hannah.christensen@bristol.ac.uk

Caroline Trotter

Department of Veterinary Medicine, University of Cambridge, UK.

clt56@cam.ac.uk

For peer review only

Martin Maiden

Department of Zoology, University of Oxford, UK.

martin.maiden@zoo.ox.ac.uk

Adam Finn

School of Population Health Sciences, Bristol Medical School, University of Bristol, UK.

adam.finn@bristol.ac.uk

Matthew D Snape

Oxford Vaccine Group, Department of Paediatrics, University of Oxford and the National Institute for Health Research (NIHR) Oxford Biomedical Research Centre, Oxford, UK.

matthew.snape@paediatrics.ox.ac.uk

and the 'Be on the TEAM' investigators (**as collaborators**):

Paul Heath

St George's University Hospital NHS Foundation Trust, UK.

pheath@sgul.ac.uk

David Turner

Centre of Biological Sciences, University of Nottingham & Nottingham Hospitals NHS Trust, UK.

david.turner@nottingham.ac.uk

Elizabeth Whittaker

Imperial College London, UK.

Imperial College Healthcare NHS Trust, London, UK .

e.whittaker@imperial.ac.uk



For peer review only - http://bmjopen.bmj.com/site/about/guidelines.xhtml

Mala Raman

Plymouth Hospitals NHS Trust, UK.

mraman@nhs.net

Stephen Hughes

Manchester University NHS Foundation Trust, UK.

Stephen.Hughes@mft.nhs.uk

Andrew Smith

College of Medical, Veterinary & Life Sciences, University of Glasgow, UK.

Andrew.Smith@glasgow.ac.uk

Claire Cameron

NHS National Services Scotland, Health Protection Scotland, UK.

clairecameron1@nhs.net

David Orr

Lancashire Teaching Hospitals NHS Foundation Trust, UK.

david.orr@lthtr.nhs.uk

David Baxter

Stockport NHS Foundation Trust, UK.

david.baxter@stockport.nhs.uk

Christopher Williams

Health Protection, Public Health Wales, Cardiff, UK.

christopher.williams25@wales.nhs.uk

For peer review only

Rohit Gowda

Maidstone and Tunbridge Wells NHS Trust, UK.

rohit.gowda1@nhs.net

Christos Zipitis

Wrighton, Wigan and Leigh NHS Foundation Trust, UK

christos.zipitis@wwl.nhs.uk

Katy Fidler

Brighton and Sussex Medical School, UK.

Royal Alexandra Children's Hospital, Brighton and Sussex University Hospital NHS Trust, Brighton, UK.

k.fidler@bsms.ac.uk

Sujata Khajuria

Northamptonshire Healthcare NHS Foundation Trust, UK.

sujata.khajuria@nhft.nhs.uk

Manuscript Word Count: 4438

For peer review only

# ABSTRACT


## Introduction

Capsular group B *Neisseria meningitidis* (MenB) is the most common cause of invasive meningococcal disease (IMD) in many parts of the world. A MenB vaccine directed against the polysaccharide capsule remains elusive due to poor immunogenicity and safety concerns. The vaccines licenced for the prevention of MenB disease, 4CMenB (Bexsero®) and MenB-fHbp (Trumenba®), are serogroup B 'substitute' vaccines, comprised of subcapsular proteins and are designed to provide protection against most MenB disease-causing strains. In many high-income countries, such as the UK, adolescents are at increased risk of IMD and have the highest rates of meningococcal carriage. Beginning in the late 1990s, immunisation of this age group with the meningococcal group C conjugate vaccine reduced asymptomatic carriage and disrupted transmission of this organism, resulting in lower group C IMD incidence across all age groups. Whether vaccinating teenagers with the novel 'MenB' protein-based vaccines will prevent acquisition or reduce duration of carriage and generate herd protection was unknown at the time of vaccine introduction and could not be inferred from the effects of the conjugate vaccines. 4CMenB and MenB-fHbp may also impact on non-MenB disease-causing capsular groups as well as commensal *Neisseria* species. This study will evaluate the impact of vaccination with 4CMenB or MenB-fHbp on oropharyngeal carriage of pathogenic meningococci in teenagers, and consequently the potential for these vaccines to provide broad community protection against MenB disease.

## Methods and Analysis

The 'Be on the TEAM' study is a pragmatic, partially-randomised controlled trial of 24000 16-19 year-old students in their penultimate year of secondary school across the UK with regional allocation to a 0+6 month schedule of 4CMenB or MenB-fHBP or to a control group. Culture-confirmed oropharyngeal carriage will be assessed at baseline and at 12 months, following which the control group will be eligible for 4CMenB vaccination. The primary outcome is the carriage prevalence of




For peer review only - http://bmjopen.bmj.com/site/about/guidelines.xhtml

potentially pathogenic meningococci (defined as those with genogroups B, C, W, Y, or X), in each vaccine group compared separately to the control group at 12 months post enrolment, i.e. 12 months after the 1st vaccine dose and 6 months after the 2nd vaccine dose. Secondary outcomes include impact on carriage of: genogroup B meningococci; hyperinvasive meningococci; all meningococci; those meningococci expressing vaccine antigens and the impact on carriage of other *Neisseria* species. A sample size of 8000 in each arm will provide 80% power to detect a 30% reduction in meningococcal carriage, assuming genogroup B, C, W, Y, or X meningococci carriage of 3.43%, a design effect of 1.5, a retention rate of 80% and a significance level of 0.05. Study results will be available in 2021 and will inform UK and international immunisation policy and future vaccine development.

## Ethics and Dissemination

This study is approved by the National Health Service (NHS) South Central Research Ethics Committee (18/SC/0055); the UK Health Research Authority (IRAS ID 239091) and the UK Medicines and Healthcare products Regulatory Agency. Publications arising from this study will be submitted to peer-reviewed journals. Study results will be disseminated in public forums, online, presented at local and international conferences and made available to the participating schools.

## Prospective Clinical Trials Registration

EudraCT: 2017-004609-42

## Keywords

*Neisseria meningitidis*. Meningococcal Infections. Vaccination. 4CMenB. MenB-fHbp.



For peer review only - http://bmjopen.bmj.com/site/about/guidelines.xhtml

# Article Summary

## Strengths and Limitations of this Study

- The scale of this carriage study will compensate for the known variation in carriage rates that can occur even when consistent methodology is used across multiple sites. The wide geographic distribution of study sites increases applicability to other settings.
- Regional allocation of schools to the same study group may increase the power to detect population effects in discrete social networks.
- The duration of post-immunisation follow up (6 months) and a single post-immunisation sampling point limits the potential to identify a longer-term or more short-lived reduction in carriage, however, the mobility of school-leavers renders longer-term follow up during the highest period of acquisition infeasible in this study.
- This study will inform modelling estimates of potential community-wide impact of adolescent immunisation with either 4CMenB or MenB-fHbp, which are dependent on whether these vaccines induce herd protection.

For peer review only

# Introduction

## Background and Rationale

Capsular group B *Neisseria meningitidis* (MenB) accounts for most cases of invasive meningococcal disease (IMD) in the United Kingdom, Europe, North America, Australia, New Zealand and many countries in Latin America.[1-3] Most IMD cases occur in infants and young children, with a smaller peak in incidence in late adolescence. Despite advances in recognition and management, the global case fatality rate remains 9%, with substantial sequelae in many survivors.[1] Whilst conjugate vaccines that target the polysaccharide capsule of groups A, C, W and Y meningococci are highly effective, a capsule-based vaccine for MenB remains elusive due to poor immunogenicity and potential autoimmunity resulting from homology with surface glycoproteins on human embryonic neural cells.[4] Consequently the vaccines licenced for the prevention of IMD due to MenB, 4CMenB (Bexsero®, GSK) and MenB-fHbp (Trumenba®, Pfizer), target subcapsular proteins. These proteins are highly diverse and have variable density of surface expression. Thus, adequate breadth of coverage relies on a multi-component strategy and potential to generate cross-protective vaccine-induced antibodies. The 4CMenB vaccine contains factor H-binding protein (fHbp) from Sub-family B / Variant 1 fused with the accessory protein GNA2091; *Neisseria* heparin binding antigen (NHBA), Neisserial adhesin A (NadA) fused with the accessory protein GNA 1030; and an outer-membrane vesicle NZ98/254 (PorA 1.4). MenB-fHbp contains two self-lipidated alleles of fHbp, one each from Sub-family A / Variant 3 and Sub-family B / Variant 1. 4CMenB and MenB-fHbp were licensed based on safety and immunogenicity data determined by human serum bactericidal antibody activity (hSBA) as an established correlate of protection.[5,6]

In September 2015, the UK introduced a routine 4CMenB infant immunisation programme, in which all infants born after the 1st of May 2015 are eligible to receive a 2+1 schedule of 4CMenB at 2, 4 and 12 months of age. Ten months after introduction, the effectiveness of the 2 and 4 month doses against



For peer review only

all MenB IMD was estimated at 83% with very broad uncertainty (95% CI 24.1-95.2), a result consistent with the estimated breadth of coverage from immunogenicity studies.[3] At three years, expected cases of MenB IMD in vaccine eligible children reduced by 75%.[7] Vaccine effectiveness of the 2+1 schedule was 59.1% (95% CI -31.1 – 97.2) against all MenB IMD and 71.2% against vaccine preventable MenB strains. There are no other vaccine effectiveness studies for either 4CMenB or MenB-fHbp.

Herd protection was not observed or expected from the UK MenB infant programme because carriage is very rare in infants as compared with the peak carriage rates that occur in late adolescence and early adulthood.[8] The potential for indirect protection to enhance vaccine impact and cost-effectiveness is exemplified by the meningococcal group C conjugate (MCC) vaccine campaign that included children, adolescents and young adults. A reduction in carriage in this targeted population dramatically reduced IMD caused by serogroup C meningococcus (MenC) in all age groups in the UK.[9] Maintaining this reduction in carriage and therefore MenC IMD in the UK is the primary rationale for an adolescent booster dose of a MenC containing vaccine in early adolescence following infant priming with a single MCC dose given at 12 months of age.

Based on modelling estimates, an infant-only MenB immunisation programme may prevent 26.3% of all-age IMD in the first five years after introduction.[10] If MenB vaccines could reduce meningococcal carriage by 30% and therefore disrupt transmission, a combined infant and adolescent immunisation programme could reduce all-age annual cases by 48.8% at 10 years and 59.7% at 20 years after introduction, enhancing impact and cost-effectiveness.[10] The impact of 4CMenB immunisation on carriage has been studied in two randomised controlled trials (RCT) to date. The 'B Part of It' cluster-randomised controlled trial assessed the impact of immunisation with 4CMenB on carriage in 24269 secondary school students in Australia.[11] There was no impact on carriage of pathogenic genogroups A, B, C, W, Y, or X at 12 months following immunisation. In the UK, 2954 university students were randomised to receive 4CMenB, MenACWY-CRM, or to an active control.[12] This study showed no effect on the primary outcome of carriage prevalence 1 month after a two-dose schedule of 4CMenB.



For peer review only - http://bmjopen.bmj.com/site/about/guidelines.xhtml

Using a composite end point of carriage at any timepoint 3 to 12 months following immunisation, administration of 4CMenB reduced carriage of any meningococcus by 18.2% (95% CI 3.4 - 30.8) and meningococcus groups B, C, W and Y combined by 26.6% (95% CI 10.5 - 39.9). This reduction was primarily due to a fall in carriage of meningococci of capsular groups C, W, and Y, with a non-significant change in capsular group B meningococcal carriage. Only a minority of students at any institution received the vaccine, which reduced the potential impact of herd protection in the cohort. Studies assessing MenB-fHbp carriage impact are limited to observational reports of several small MenB outbreaks in US universities,[13] where a number of interventions including mass-immunisation campaigns halted new cases, however these studies were not specifically designed or powered to show an impact of MenB-fHbp vaccine on carriage.

4CMenB and MenB-fHBP may elicit immune responses against non-MenB meningococci and, for 4CMenB, *Neisseria gonorrhoeae.*[14,15] Vaccine antigens may also be present in commensal *Neisseria* species resident in the oropharynx.[15,16] Whilst cross-protection against meningococci expressing other disease-associated capsular groups is desirable, a broader impact on colonising strains of *N. meningitidis* or potentially protective non-invasive organisms such as *Neisseria lactamica* requires surveillance. Unencapsulated meningococci, which are generally non-pathogenic, may have greater surface exposure of vaccine antigens and therefore be more vulnerable to mucosal clearance than the potentially invasive capsule-expressing meningococci. Natural exposure to these bacteria throughout childhood contributes to the development of immunity against hyper-invasive strains.[17] It is not known whether vaccines that induce immunity to these *Neisseria* species may facilitate an unintended niche for pathogenic meningococci.


For peer review only - http://bmjopen.bmj.com/site/about/guidelines.xhtml

# Methods

## Objectives and Study Design

The 'Be on the TEAM' (Teenagers Against Meningitis) study is a pragmatic, partially-randomised cluster controlled trial that will evaluate the carriage prevalence of pathogenic meningococci in adolescents vaccinated with either 4CMenB or MenB-fHbp, compared with a control group. Participants aged 16 - 19 years will be enrolled in 16 regions across the UK, with each region allocated to: (i) immunisation at 0 and 6 months with 4CMenB ("4CMenB Group"); (ii) immunisation at 0 and 6 months with MenB-fHbp ("MenB-fHbp Group"); or (iii) an unimmunised Control Group who will be eligible for deferred vaccination with 4CMenB. Oropharygneal swab samples (OPSs) will be taken at baseline and at 12 months.

The primary objective is to describe the carriage prevalence of culture-confirmed *N. meningitidis* genogroup B, C, W, Y, X (i.e. those meningococci possessing genes encoding the capsular polysaccharides corresponding to the respective serogroups) at 12 months after baseline vaccination (6 months following the second vaccine dose) in the 4CMenB and MenB-fHbp Groups compared with the unimmunised Control Group. The study will not directly compare the 4CMenB Group with the MenB-fHbp Group.

The secondary objectives are to determine whether immunisation with either 4CMenB or MenB-fHbp affects the carriage prevalence of:

- Genogroup B meningococci;
- Meningococci of other genogroups and capsule null meningococci;
- Strains belonging to hyper-invasive meningococcal lineages;


For peer review only - http://bmjopen.bmj.com/site/about/guidelines.xhtml

- All meningococcal strains;
- Meningococci expressing antigens contained in MenB-fHbp or 4CMenB;
- Other *Neisseria* species.

A further secondary objective will be to determine any difference in rates of new acquisition of carriage between groups as defined in the primary and secondary objectives above.

As a stand-alone exploratory objective, this study will also permit an assessment of the UK adolescent quadrivalent MenACWY vaccination campaign on carriage.[18] This campaign was introduced in 2015 due to a rise in capsular group W meningococcal (MenW) disease and has high coverage in the school population of prospective participants in this study. "UKMenCar4", an adolescent carriage study across the same study network in 2014/15 will act as the pre-intervention study. The post-intervention carriage prevalence will be determined by the baseline carriage samples from the Be on the TEAM study.[19]

## Study setting

This is a multi-centre, school-based study in 16 regions across the UK (Figure 1). Students will be aged 16-19 years at time of enrolment, attending school or college Year 12 (in England and Wales) or S5 (in Scotland), or their equivalent. This will include both government (local authority) and independent schools, 6th form colleges, single sex or coeducation schools, boarding or day schools. Study visits will be conducted in schools, although follow-up visits may occur in a community setting if participants have left school.



For peer review only - http://bmjopen.bmj.com/site/about/guidelines.xhtml

## Sample Size

The sample size of 24 000 participants (8000 in each arm) is based on an estimated carriage rate 3.43% for genogroup B, C, W, Y or X in the control group at the 12 month swab. When assuming a retention rate of 80% and a design effect of 1.5, this sample size will provide 80% power to detect a 30% reduction of these genogroups combined, at a significance level of 0.05.

This anticipated carriage rate is based on results from the UKMenCar4 study, which determined carriage prevalence of 1.65% for genogroup B meningococcus, 2.3% for hyper-invasive meningococci and a combined prevalence of 4.2% for genogroup B, C, W, Y, and X meningococci.[19] The figure of 3.43% accounts for a potential 50% reduction in carriage of capsular groups W and Y due to the UK quadrivalent MenACWY vaccination programme; this figure is necessarily an estimate as there are limited data on the impact of the universal adolescent quadrivalent ACWY vaccine campaign on carriage. A previous study in UK university students showed that immunisation with MenACWY-CRM reduced carriage of genogroup C, W or Y meningococci by 27.1% (95% CIs 6.9 - 42.9) and serogroup C, W, or Y by 36.2% (95% CIs 15.6 - 51.7).[12] A further UK university carriage cohort study suggested that the quadrivalent MenACWY vaccine exerts differential effects on capsular groups and did not prevent expansion of meningococci belonging to the hyperinvasive genogroup W clonal complex 11, although this was based on cross sectional surveys rather than a longitudinal design.[20]

## Study Timeline

In order to meet recruitment targets and maximise the interval between immunisation and the final OPS within the structure of the school exam year, the study will recruit across 4 "waves" over 20 months: Wave 1 March-May 2018; Wave 2 September-November 2018; Wave 3 March-May 2019; Wave 4 September-November 2019 (Figure 2). The academic teaching year runs from September until



For peer review only - http://bmjopen.bmj.com/site/about/guidelines.xhtml

April or May, depending on local jurisdictions. Recruitment will be balanced between study groups for each of the 'Spring' (March to May) and 'Autumn' (September to November) recruiting periods in view of expected seasonal variation in carriage. Completion of the final post-vaccination pharyngeal swab collection is expected in early 2021.

## Eligibility Criteria

Participants will be healthy adolescents aged 16 - 19 years old at time of enrolment, in their penultimate year of participating secondary school or 6th form colleges, who intend to return for a further year of school. Students will be eligible for enrolment if they are willing and able to provide informed consent and meet the additional inclusion criteria:

- willing to comply with all study requirements;
- consent for storage of oropharyngeal swabs and any bacterial isolates for future research;
- willing to allow their General Practitioner to be contacted to confirm vaccination status or medical history.

Participants will be ineligible to participate if they have received a course of either 4CMenB or MenB-fHbp in the past (by documentation or self-report). Furthermore, participants in the 4CMenB Group or MenB-fHbp Group will be ineligible if they meet any of the following criteria:

a. History of anaphylaxis to any component of 4CMenB or MenB-fHbp (For 4CMenB only, this includes allergy to latex);
b. Any other significant disease or disorder, which in the opinion of the investigator may: put the participants at risk because of participation in the trial, influence the result of the trial or affect the participant's ability to participate. Specific examples include haemophilia or medically diagnosed bleeding disorder, or receipt of anticoagulant medication, that prohibits the use of intramuscular injections

For peer review only

c. known or suspected pregnancy.

Participants in the Control Group that fail any of the specific exclusion criteria *a, b* or *c* will be eligible to participate in the assessment of carriage, but will not be able to receive the 4CMenB vaccine after the final OPS. Ineligibility arising during the trial due to criteria *a*, *b* or *c* in the 4CMenB Group or MenB-fHbp Group will allow inclusion for assessment of carriage at the study endpoint but will exclude a participant from vaccination.

# Study Procedures

For peer review only

## Recruitment

Recruitment will occur in participating schools; individual participants will not be directly recruited by the study teams outside of the school setting. Social media and the study website (www.beontheteam.uk ) will be used for study information and awareness, but these platforms will not be used for recruitment. Participants will receive information through a school assembly about meningococcal disease and the study rationale and design. Subsequently, they will receive a written participant information sheet.

## Informed Consent

All participants will be aged 16 years or over, and are therefore self-consenting as per the National Institute of Health Research guidelines.[21] However, parents/guardians will be provided with information about the study via school communication platforms as well as directly by prospective participants. Consent will be taken by clinical staff (registered doctor or nurse) or non-clinical staff who have had appropriate experience and training. Where interested participants have vulnerabilities that may impair their capacity to provide informed consent, additional input or support from the


For peer review only - http://bmjopen.bmj.com/site/about/guidelines.xhtml

individual's parents/guardians will be sought. If there is ongoing doubt about an individual's ability to provide informed consent, then they will not be enrolled in the study.

## Randomisation, Allocation and Blinding

This is an open-label partially-randomised study, with group allocation by study site. All schools within the geographical coverage area of each study site will be allocated to the same study arm, both for logistical reasons and because geographic allocation may increase the potential to detect any impact on herd protection. As a post-licensure study, blinded administration and/or placebo will not impact on the outcome of carriage prevalence and would introduce unnecessary cost and complexity to a trial of this scale. The allocation of initial sites was determined prior to the start of the study (Figure 1). A capacity assessment stratified sites to either the control arm or immunisation sites depending on existing site expertise and the requirements to maintain equal numbers in each study arm. The immunisation sites were randomised to either the MenB-fHbp Group or the 4CMenB Group. Additional sites may be added during the study and will be pragmatically allocated to a study group to ensure balanced recruitment.

## Interventions

All participants will be required to attend three study visits at their school (Figure 3). Following informed consent, a baseline OPS will be taken by study staff using a standardised method of collection. A flocked swab will be used to sweep from one tonsil across the posterior pharynx to the contralateral tonsil. Participants in all groups will complete a brief questionnaire collecting demographic data and risk factors for meningococcal carriage such as smoking and antimicrobial use (see supplementary file).[22] The questionnaire is compatible with previous large-scale UK meningococcal carriage studies conducted in 1999, 2000, 2001, and 2014/15, and the B Part of It Australian study, which will allow an assessment of trends over time.[11,19,23,24] The 4CMenB and MenB-

For peer review only

fHbp groups will both receive a two dose vaccine schedule at 0 and 6 months and all groups will have a second OPS taken at 12 months following enrolment. The Control Group will be offered a two dose schedule of 4CMenB vaccination after their second OPS. Individual swab results will not be given to participants, in accordance with previous meningococcal carriage studies, however participants will be contacted confidentially if *Neisseria gonorrhoeae* is detected.

## Public and Patient Involvement

This study will provide a unique opportunity to engage adolescents on an unprecedented scale to: (i) increase awareness of meningococcal disease; (ii) highlight the importance of immunisation; and, (iii) stimulate an interest in involvement in impact-driven research that potentially benefits the broader community. All Year 12 (or equivalent) students at participating schools will attend assemblies, often involving meningitis survivors and disease prevention advocates. These 'meningitis ambassadors' will discuss their personal experience of meningitis and provide education about signs and symptoms of meningitis. An ethically approved presentation will describe the science behind the study, including information about IMD and the link between vaccination, carriage and herd protection. Communication material used in this study will be developed with input from focus groups run by two meningitis charities, Meningitis Now and the Meningitis Research Foundation, who will also coordinate the provision of 'meningitis ambassadors'. Other initiatives include a social media policy engaging students, schools and research teams with curated social media posts to complement public engagement aims. Combining these strategies will ensure that participants make a fully-informed decision to participate in the study. Furthermore, by involving entire school year cohorts (regardless of enrolment into the study) an estimated 48 000 students across 200 schools will be engaged in a critical conversation about meningitis, research and the benefits of immunisation across the broader community. Study results will be submitted for publication in peer-reviewed journals, and then



For peer review only - http://bmjopen.bmj.com/site/about/guidelines.xhtml

disseminated directly to the schools involved, as well as on the study social media platforms, and the Meningitis Now and Meningitis Research Foundation websites.

## Equality and Diversity

The study will be done across 16 regions throughout the UK and will include schools across the range of socioeconomic strata. School types reflect the variety of educational institutions across the UK as detailed in the study setting. Previous diversity information collected as part of UK MenCar4 across the same study network demonstrated that participants were reflective of their communities according to national census data.[25] Inclusion of whole schools, rather than targeting individuals, will encourage equal participation of diverse populations.

## Sample Handling and Laboratory Processes

Collected OPS will be placed immediately into STGG broth (skim milk, tryptone, glucose, glycerine; EO Labs, Bonnybridge, UK), maintained at 2-8$^0$C until frozen at -80$^0$C, within 4 hours of collection (figure 4). Site laboratories will thaw and plate the OPS sample on selective GC-VCAT agar (vancomycin, colistin amphotericin B, trimethoprim; Thermo Fisher Scientific, Basingstoke, UK) at 37$^0$C in 5% $CO_2$ for up to 48 hours. Putative *Neisseria* species (oxidase positive, Gram-negative diplococci) will be subcultured on to Columbia Horse Blood Agar, then stored in a bead-based cryovial (Technical Service Consultants, Lancashire, UK) at -80$^0$C.

The Public Health England Meningococcal Reference Unit (Manchester), will serogroup putative *Neisseria* isolates using an in-house dot-blot ELISA using NIBSC monoclonal antibodies against capsular polysaccharide serogroups B, C, W and Y.[26] DNA will be extracted using a bead-based MagMAX DNA Multi-Sample Ultra Kit (Thermo Fisher Scientific, MA, USA). Total DNA extracts will be normalised prior



For peer review only - http://bmjopen.bmj.com/site/about/guidelines.xhtml

to whole genome sequencing on the Illumina NovaSeq6000 platform (Oxford Genomic Centre, Wellcome Trust Centre for Human Genetics, University of Oxford).

Genomes will be assembled using an in-house assembly pipeline (Department of Zoology, University of Oxford) which incorporates Velvet and VelvetOptimiser.[27,28] Assembled draft genome sequences will then be submitted to the open-access online database http://pubMLST.org/neisseria, with isolate providence data and FASTQ files uploaded to the European Nucleotide Archive and the Sequence Read Archive databases. Automated locus annotation using the BIGSdb software platform will be used concurrently with manual curation for classification of isolates including species, clonal complex, lineage, genogroup, and meningococcal typing schemes for multilocus sequence typing (MLST), ribosomal MLST, core genome MLST, and vaccine antigen variants (e.g. Bexsero Antigen Sequence Type, BAST).[29,30] After study completion, genomes will be published open access on the pubMLST.org/neisseria database and OPSs*,* *Neisseria* spp. isolates and any products of cultureon GC-VCAT media will be stored in an approved facility for further research.

## Potential benefits and harms

Participants will benefit from immunisation with a licenced 'MenB' vaccine which they would not otherwise receive in the UK immunisation schedule. Prior to enrolment, participants will receive information about 4CMenB and MenB-fHbp vaccination, according to the licenced product information. As a participant engagement exercise, all participants completing the final swabbing visit will be entered into a prize-draw for headphones. Participants will not receive payments.



For peer review only - http://bmjopen.bmj.com/site/about/guidelines.xhtml

## Safety Reporting and Study Oversight

This study involves products used according to their licences. Safety monitoring will focus on detecting any serious adverse events (SAEs) and suspected unexpected serious adverse reactions (SUSARs). Specific SAE exemptions include traumatic injuries; hospital admissions for intoxication, deliberate self-harm or elective management of pre-existing conditions unless in response to a worsening of that condition since study enrolment. All SAEs will be reported to the Chief Investigator and to the Sponsor, the University of Oxford Clinical Trials and Research Group. The Oxford University Hospitals NHS Foundation Trust / University of Oxford Trials Safety Group will review all new SAEs on a quarterly basis. SUSARs will be reported by the study Chief Investigator to the relevant Competent Authority and to the Research Ethics Committee. The lead site is the Oxford Vaccine Group, Department of Paediatrics, University of Oxford. The Scientific Advisory Board will provide input into study design, statistical analysis and will oversee safety reporting and study conduct in accordance with Good Clinical Practice. The Oxford Vaccine Group on behalf of the Sponsor will act as the study monitor.

## Statistical Methods

A baseline analysis will occur after Wave 2 when the study reaches approximately 50% enrolment. This descriptive analysis of baseline carriage rates will assess the validity of the prospective sample size calculation. There will be no interim analysis of vaccine impact. The principal analysis will be conducted using a modified intention to treat (mITT) approach, including all participants who have had two throat swabs. Participants will be analysed according to allocated group, regardless of vaccine receipt. Based on previous UK school-based meningococcal carriage studies, the design effect was assumed to be 1.5 in the sample size calculations but will be calculated on the actual data collected.[23,24]

For peer review only

For the primary objective and each of the secondary objectives, carriage rates will be expressed as a mean with two-sided 95% Clopper-Pearson confidence intervals for each group at baseline and at 12 months. The carriage rates in the Men-fHbp and 4CMenB groups will be compared separately to the Control Group. The odds ratio (OR) with two-sided 95% confidence intervals will be calculated using logistic regression via a generalised estimating equations (GEE) model using an exchangeable working correlation structure. Model covariates will include age, gender, season of recruitmentand baseline carriage status *a priori*. Additional risk factors found to affect carriage at baseline will also be included. Gender and smoking (smoking, home smoking and vaping) are known to effect both vaccine-induced immune responses and meningococcal carriage and these interactions will be reported regardless of significance. The level of statistical significance will be 0.05. There will be no imputation of missing data for the principal analysis.

Sensitivity analyses will be done for the primary and each of the secondary objectives and will include: analyses without adjustment or inclusion of covariates for completeness and to confirm the results from the primary analyses; a per-protocol analysis of participants who received both vaccine doses in the 4CMenB and MenB-fHbp groups; adjustment for the month of the outcome measure rather than season of recruitment to further investigate the potential effect of seasonality and; multivariable imputation for missing data. All variables that will be included in the analysis model will be included in the imputation model and will assume data are missing at random.

## Data Collection and Management

Informed consent forms will be completed on paper. Source data entry will be on a standard case report form, either paper based, or direct electronic entry using REDCap, a browser-based electronic data capture record, hosted by the University of Oxford.[31] Participant data will be handled in accordance with Good Clinical Practice and the General Data Protection Act. Participants will consent


For peer review only - http://bmjopen.bmj.com/site/about/guidelines.xhtml

to the storage of de-identified OPSs, isolates and questionnaire data. All identifiable data will be destroyed after the youngest participant in the trial turns 21 years.

## Discussion

This is the first large-scale adolescent meningococcal carriage study to assess the impact of both the 4CMenB and MenB-fHbp vaccines on carriage of pathogenic meningococci as well as commensal *Neisseria* species. Building on an established meningococcal carriage and vaccine clinical trial network, the backwards compatibility of this study will allow comparisons with four previous carriage studies in 1999, 2000, 2001, and 2014/15. This study will also facilitate an evaluation of the UK adolescent quadrivalent MenACWY vaccination program on carriage since its introduction in 2015. The 16 sites across the UK represent a geographically and demographically diverse population, increasing applicability to broad settings.

The 'B part of It' trial showed that there is no significant difference in carriage of genogroup A, B, C, W, Y and X meningococci between adolescents who received 4CMenB compared with controls.[11] Overall, the carriage of genogroup B meningococcus was low. The 'B Part of It' study has a similar design as a school-based study of 16-18 year olds; however, there are some notable difference between the design of the B Part of It study and the present study, specifically, the 'Be on the TEAM' study:

(i) includes both MenB-fHBP and 4CMenB;

(ii) allocates vaccine arms on a regional basis rather than cluster randomisation at the level of schools, which may increase the potential to observe herd immunity;

(iii) uses a 0 and 6 month vaccine schedule, longer than the 1-2 month interval used in the B Part of It study;


For peer review only - http://bmjopen.bmj.com/site/about/guidelines.xhtml

(iv) has a culture-defined endpoint of carriage, compared with direct PCR amplification targeting PorA in the B Part of It Study.

Despite the difference in study designs, cross-study collaborations will enrich understanding of the potential vaccine impact on specific *N. meningitidis* strains and commensal *Neisseria* species by genome level interrogation.

The short follow-up period of between 6 to 9 months following the second vaccination is a limitation of this study, reducing the ability to observe any potential impact during later periods of high-acquisition risk, e.g. when commencing tertiary education.[32] The large sample size required and mobility of school-leavers limits the feasibility of ongoing carriage surveillance. The secondary endpoint assessing the impact on other *Neisseria* species is based on those present in the oropharynx and culturable on selective media, however all swabs and products of culture will be stored for potential further analysis.

Due for completion in 2021, The 'Be on the TEAM' study will directly inform international MenB immunisation policy. It will contribute to an open-access repository of *Neisseria* spp. genomes and provide a biobank of samples to assist in identifying strategies for future MenB vaccine development.

## Current Study Progress

At the time of writing 24 048 participants have been enrolled from over 120 schools across the UK. Assemblies raising awareness about meningitis, immunisation and research have reached over 40,000 students. In March 2020, study visits were temporarily suspended due to the SARS-CoV-2 pandemic and at this time, 11 170 participants had successfully completed the study. The protocol may require modifications to fulfil the study objectives.


For peer review only - http://bmjopen.bmj.com/site/about/guidelines.xhtml

## Acknowledgments

The Scientific Advisory Board: Chair – Sir Brian Greenwood; Deputy Chair – James Stuart; Simon Nadel; Helen Marshall; Helen Bedford; Nick Andrews. Holly Bratcher and Odile Harrison, Department of Zoology, University of Oxford; Melanie Carr, Karen Ford, Hannah Roberts, Yama Farooq, Simon Kerridge and Annabel Giddings, Department of Paediatrics, University of Oxford; Shari Sapuan, St George's University NHS Foundation Trust; Rebecca Ramsay Brighton and Sussex University Hospitals NHS Foundation Trust; Dr Katrina Cathie, NIHR Southampton Clinical Research Facility and Department of Paediatrics, University Hospital Southampton NHS Foundation Trust. The authors acknowledge Meningitis Now and Meningitis Research Foundation for their input into study branding and design, educational material and organising meningitis ambassadors.

## Author Contributions

The study protocol was developed by MS, AF, MM, CT, HC, RB, SG, JM, PH, SF, KD, SC, PA, EP, JM, JC. CT and HC provided statistical expertise. The manuscript was drafted by JC and MS with input and editing from all authors and collaborators. All authors and collaborators reviewed the final manuscript and approved its contents.

## Funding

This study is independent research funded by the National Institute for Health Research (NIHR) Policy Research Programme ("Be on the TEAM", "Evaluating the effect of 'MenB' vaccination on meningococcal carriage", PR-R18-0117- 21001). Matthew Snape and the Oxford Vaccine Group, Department of Paediatrics, are supported by the NIHR Oxford Biomedical Research Centre. The views expressed in this publication are those of the authors and not necessarily those of the NHS, the NIHR or the Department of Health and Social Care. MenB-fHbp donated by Pfizer. Meningitis Now provided funding for a prize draw to facilitate participant retention. The funders have no role in the study design, conduct, analysis or publications arising from this study.



For peer review only - http://bmjopen.bmj.com/site/about/guidelines.xhtml

## Competing Interests

Nothing to disclose: JC; EP; JM; KD; MM; SG. AF reports grants from the Department of Health, during the conduct of the study; grants from Pfizer, grants from GSK, outside the submitted work. HC reports other from Sanofi Pasteur, other from IMS Health, grants from National Institute for Health Research, other from AstraZeneca , grants and other from GSK, outside the submitted work. MS reports grants from National Institute for Health Research (NIHR), non-financial support from Pfizer, during the conduct of the study; grants from Pfizer, grants from MCM, grants from GlaxoSmithKline, grants from Novavax, grants from Medimmune, grants from Janssen, outside the submitted work. SC reports salary contribution from the study NIHR grant, during the conduct of the study. SF reports personal fees were paid to his institution (with no personal payment of any kind) from AstraZeneca/Medimmune, Sanofi, Pfizer, Seqirus, Sandoz, Merck, GSK, Alios, Johnson & Johnson, and Merck, outside the submitted work. RB reports grants from University of Oxford via a NIHR grant, during the conduct of the study; contract research on behalf of Public Health England for GSK, Pfizer and Sanofi Pasteur, outside the submitted work.

## Approvals & Clinical Trials Registration

National Health Service (NHS) South Central Research Ethics Committee (18/SC/0055). The UK Health Research Authority (IRAS ID 239091). UK Medicines and Healthcare products Regulatory Agency. Prospective Clinical Trials Registration EudraCT: 2017-004609-42.

## Protocol

Current Protocol Version 5.0 dated 24-Jan-2020. All protocol modifications will be reported promptly to the NIHR, Department of Health and Human Services, the Scientific Advisory Board and the Principal Investigators. This report was written using the SPIRIT checklist.[33] The study protocol,

For peer review only

patient information sheets and an example consent form are publicly available on the study website: www.beontheteam.uk

## Dissemination and Authorship Policy

Publications arising from this study will be submitted to peer-reviewed journals and authorship predetermined according to ICMJE guidelines. Study results will be disseminated in public forums, online, presented at local and international conferences and made available to the participating schools.

## Data Sharing Statement

De-identified datasets will be available on reasonable request from the Chief Investigator. Genomes will be made publicly available on the open access http://pubMLST.org/neisseria database following publication of the study results.

## References

1. Sridhar S, Greenwood B, Head C, et al. Global incidence of serogroup B invasive meningococcal disease: a systematic review. *Lancet Infect Dis* 2015;15(11):1334-46. doi: 10.1016/S1473-3099(15)00217-0.
2. Peterson ME, Li Y, Bita A, et al. Meningococcal serogroups and surveillance: a systematic review and survey. *J Glob Health* 2019;9(1):010409. doi: 10.7189/jogh.09.010409.
3. Parikh SR, Andrews NJ, Beebeejaun K, et al. Effectiveness and impact of a reduced infant schedule of 4CMenB vaccine against group B meningococcal disease in England: a national observational cohort study. *The Lancet* 2016;388(10061):2775-82. doi: 10.1016/s0140-6736(16)31921-3.
4. Finne J, Leinonen M, Makela PH. Antigenic similarities between brain components and bacteria causing meningitis. Implications for vaccine development and pathogenesis. *Lancet* 1983;2(8346):355-7.
5. McIntosh ED, Broker M, Wassil J, et al. Serum bactericidal antibody assays - The role of complement in infection and immunity. *Vaccine* 2015;33(36):4414-21. doi: 10.1016/j.vaccine.2015.07.019.
6. Goldschneider I. Human Immunity to the Meningococcus: I. The Role of Humoral Antibodies. *Journal of Experimental Medicine* 1969;129(6):1307-26. doi: 10.1084/jem.129.6.1307.
7. Ladhani SN, Andrews N, Parikh SR, et al. Vaccination of Infants with Meningococcal Group B Vaccine (4CMenB) in England. *N Engl J Med* 2020;382(4):309-17. doi: 10.1056/NEJMoa1901229.
8. Christensen H, May M, Bowen L, et al. Meningococcal carriage by age: a systematic review and meta-analysis. *The Lancet Infectious Diseases* 2010;10(12):853-61. doi: 10.1016/s1473-3099(10)70251-6.

For peer review only

9. Trotter CL, Maiden MC. Meningococcal vaccines and herd immunity: lessons learned from serogroup C conjugate vaccination programs. *Expert Rev Vaccines* 2009;8(7):851-61. doi: 10.1586/erv.09.48.
10. Christensen H, Hickman M, Edmunds WJ, et al. Introducing vaccination against serogroup B meningococcal disease: an economic and mathematical modelling study of potential impact. *Vaccine* 2013;31(23):2638-46. doi: 10.1016/j.vaccine.2013.03.034.
11. Marshall HS, McMillan M, Koehler AP, et al. Meningococcal B Vaccine and Meningococcal Carriage in Adolescents in Australia. *N Engl J Med* 2020;382(4):318-27. doi: 10.1056/NEJMoa1900236.
12. Read RC, Baxter D, Chadwick DR, et al. Effect of a quadrivalent meningococcal ACWY glycoconjugate or a serogroup B meningococcal vaccine on meningococcal carriage: an observer-blind, phase 3 randomised clinical trial. *The Lancet* 2014;384(9960):2123-31. doi: 10.1016/s0140-6736(14)60842-4.
13. Soeters HM, Whaley M, Alexander-Scott N, et al. Meningococcal Carriage Evaluation in Response to a Serogroup B Meningococcal Disease Outbreak and Mass Vaccination Campaign at a College-Rhode Island, 2015-2016. *Clin Infect Dis* 2017;64(8):1115-22. doi:10.1093/cid/cix091.
14. Rodrigues CMC, Lucidarme J, Borrow R, et al. Genomic Surveillance of 4CMenB Vaccine Antigenic Variants among Disease-Causing Neisseria meningitidis Isolates, United Kingdom, 2010-2016. *Emerg Infect Dis* 2018;24(4):673-82. doi: 10.3201/eid2404.171480.
15. Petousis-Harris H, Paynter J, Morgan J, et al. Effectiveness of a group B outer membrane vesicle meningococcal vaccine against gonorrhoea in New Zealand: a retrospective case-control study. *Lancet* 2017;390(10102):1603-10. doi: 10.1016/S0140-6736(17)31449-6.
16. Lucidarme J, Gilchrist S, Newbold LS, et al. Genetic distribution of noncapsular meningococcal group B vaccine antigens in Neisseria lactamica. *Clin Vaccine Immunol* 2013;20(9):1360-9. doi: 10.1128/CVI.00090-13.
17. Pollard AJ, Frasch C. Development of natural immunity to Neisseria meningitidis. *Vaccine* 2001;19(11-12):1327-46.
18. Campbell H, Edelstein M, Andrews N, et al. Emergency Meningococcal ACWY Vaccination Program for Teenagers to Control Group W Meningococcal Disease, England, 2015-2016. *Emerg Infect Dis* 2017;23(7):1184-87. doi: 10.3201/eid2307.170236.
19. Maclennan JM, Maiden MC, Group UMC. UKMENCAR4: A meningococcal carriage study in 21,000 teenagers to understand changing meningococcal epidemiology and evaluate National vaccination policy. International Pathogenic Neisseria Conference; 2016 4th-9th September; Manchester, UK.
20. Oldfield NJ, Green LR, Parkhill J, et al. Limited Impact of Adolescent Meningococcal ACWY Vaccination on Neisseria meningitidis Serogroup W Carriage in University Students. *J Infect Dis* 2018;217(4):608-16. doi: 10.1093/infdis/jix596.
21. Choices NHS. Consent to treatment - Children and young people - NHS Choices: Department of Health; 2016 [updated 2016-28-11. Available from: http://www.nhs.uk/Conditions/Consent-to-treatment/Pages/Children-under-16.aspx.
22. MacLennan J, Kafatos G, Neal K, et al. Social behavior and meningococcal carriage in British teenagers. *Emerg Infect Dis* 2006;12(6):950-7.
23. Maiden MC, Ibarz-Pavon AB, Urwin R, et al. Impact of meningococcal serogroup C conjugate vaccines on carriage and herd immunity. *J Infect Dis* 2008;197(5):737-43. doi:10.1086/527401.
24. Maiden MC, Stuart JM, Group UKMC. Carriage of serogroup C meningococci 1 year after meningococcal C conjugate polysaccharide vaccination. *Lancet* 2002;359(9320):1829-31. doi: 10.1016/S0140-6736(02)08679-8.
25. Bratcher HB, Rodrigues CMC, Finn A, et al. UKMenCar4: A cross-sectional survey of asymptomatic meningococcal carriage amongst UK adolescents at a period of low invasive meningococcal disease incidence. *Wellcome Open Res* 2019;4:118. doi:10.12688/wellcomeopenres.15362.2.
26. Public Health England Meningococcal Reference unit User Manual November 2018. Available online



For peer review only - http://bmjopen.bmj.com/site/about/guidelines.xhtml

https://assets.publishing.service.gov.uk/government/uploads/system/uploads/attachment_data/file/753941/Meningococcal_reference_unit_user_manual.pdf accessed 05-05-2019.
27. Zerbino DR. Using the Velvet de novo assembler for short-read sequencing technologies. *Curr Protoc Bioinformatics* 2010;Chapter 11:Unit 11 5. doi: 10.1002/0471250953.bi1105s31.
28. Zerbino DR, Birney E. Velvet: algorithms for de novo short read assembly using de Bruijn graphs. *Genome Res* 2008;18(5):821-9. doi: 10.1101/gr.074492.107.
29. Jolley KA, Maiden MC. BIGSdb: Scalable analysis of bacterial genome variation at the population level. *BMC Bioinformatics* 2010;11:595. doi: 10.1186/1471-2105-11-595.
30. Bratcher HB, Corton C, Jolley KA, et al. A gene-by-gene population genomics platform: de novo assembly, annotation and genealogical analysis of 108 representative Neisseria meningitidis genomes. *BMC Genomics* 2014;15:1138. doi: 10.1186/1471-2164-15-1138.
31. Harris PA, Taylor R, Thielke R, et al. Research electronic data capture (REDCap)--a metadata-driven methodology and workflow process for providing translational research informatics support. *J Biomed Inform* 2009;42(2):377-81. doi: 10.1016/j.jbi.2008.08.010.
32. Jeppesen CA, Snape MD, Robinson H, et al. Meningococcal carriage in adolescents in the United Kingdom to inform timing of an adolescent vaccination strategy. *J Infect* 2015;71(1):43-52. doi: 10.1016/j.jinf.2015.02.006.
33. Chan AW, Tetzlaff JM, Altman DG, et al. SPIRIT 2013 statement: defining standard protocol items for clinical trials. *Ann Intern Med* 2013;158(3):200-7. doi: 10.7326/0003-4819-158-3-201302050-00583.

For peer review only

**Figure headings and legends**

**Figure 1** Study sites and vaccine group allocation

**Figure 2** Study timeline by recruitment wave and Year 12 / S5 cohort (penultimate year of secondary school) at the time of enrolment. Vaccine groups receive either MenB-fHbp or 4CMenB at 0 + 6 months. See figure 1 for visit window ranges.

**Figure 3** Study design

**Figure 4** Laboratory Analysis Plan. GC-VCAT selective media (vancomycin, colistin, amphotericin B, trimethoprim); MLST multilocus sequence typing; rMLST ribosomal MLST, cgMLST core genome MLST, cc clonal complex

For peer review only

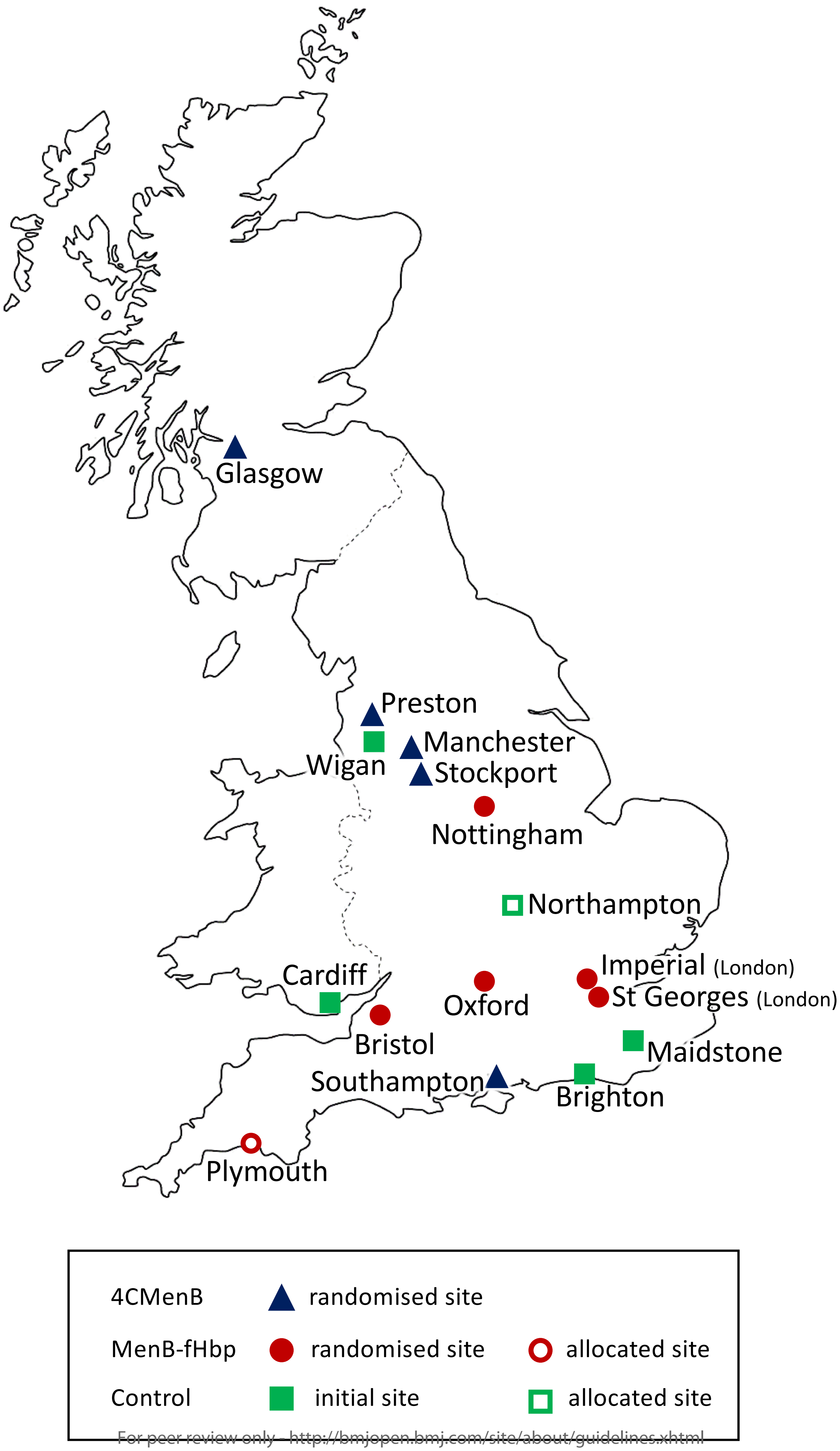


For peer review only - http://bmjopen.bmj.com/site/about/guidelines.xhtml

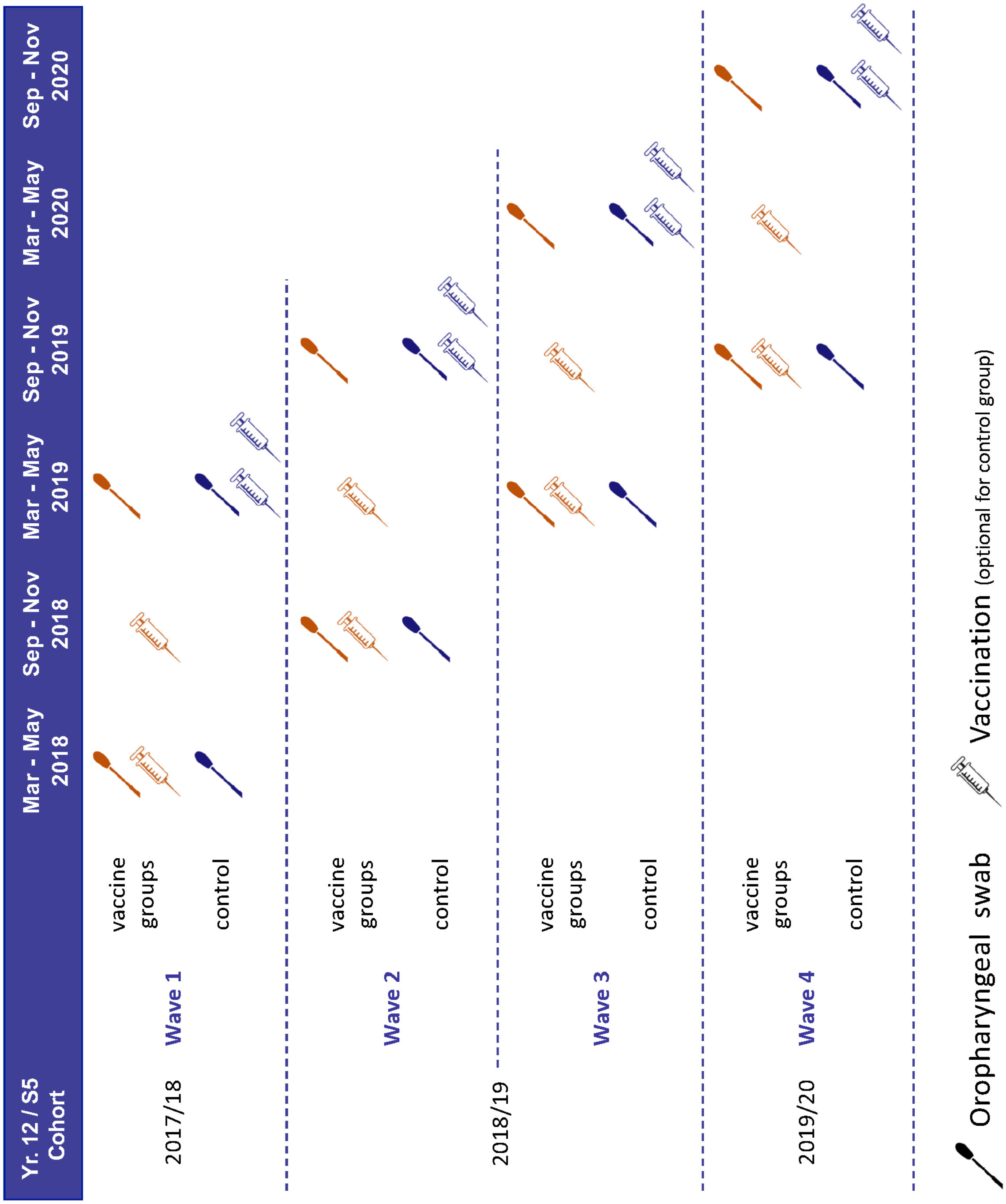
Yr. 12 / S5 Cohort
Mar - May 2018
Sep - Nov 2018
Mar - May 2019
Sep - Nov 2019
Mar - May 2020
Sep - Nov 2020
2017/18
Wave 1
vaccine groups
control
Wave 2
vaccine groups
control
2018/19
Wave 3
vaccine groups
control
2019/20
Wave 4
vaccine groups
control
Oropharyngeal swab
Vaccination (optional for control group)

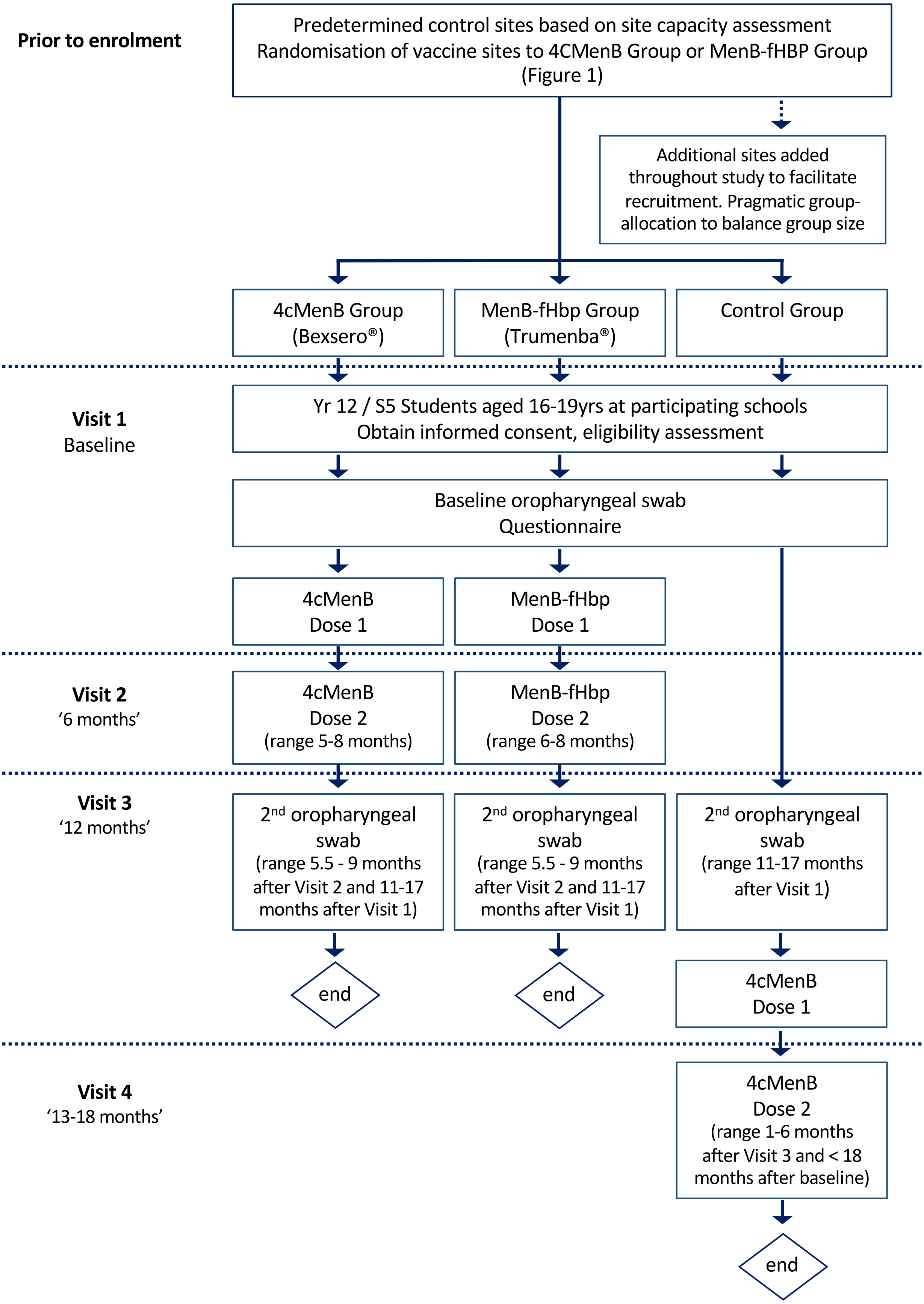
Prior to enrolment
Predetermined control sites based on site capacity assessment
Randomisation of vaccine sites to 4CMenB Group or MenB-fHBP Group
(Figure 1)
Additional sites added throughout study to facilitate recruitment. Pragmatic group-allocation to balance group size
4cMenB Group
(Bexsero®)
MenB-fHbp Group
(Trumenba®)
Control Group
Visit 1
Baseline
Yr 12 / S5 Students aged 16-19yrs at participating schools
Obtain informed consent, eligibility assessment
Baseline oropharyngeal swab
Questionnaire
4cMenB
Dose 1
MenB-fHbp
Dose 1
Visit 2
'6 months'
4cMenB
Dose 2
(range 5-8 months)
MenB-fHbp
Dose 2
(range 6-8 months)
Visit 3
'12 months'
2nd oropharyngeal swab
(range 5.5 - 9 months after Visit 2 and 11-17 months after Visit 1)
2nd oropharyngeal swab
(range 5.5 - 9 months after Visit 2 and 11-17 months after Visit 1)
2nd oropharyngeal swab
(range 11-17 months after Visit 1)
end
end
4cMenB
Dose 1
Visit 4
'13-18 months'
4cMenB
Dose 2
(range 1-6 months after Visit 3 and < 18 months after baseline)
end

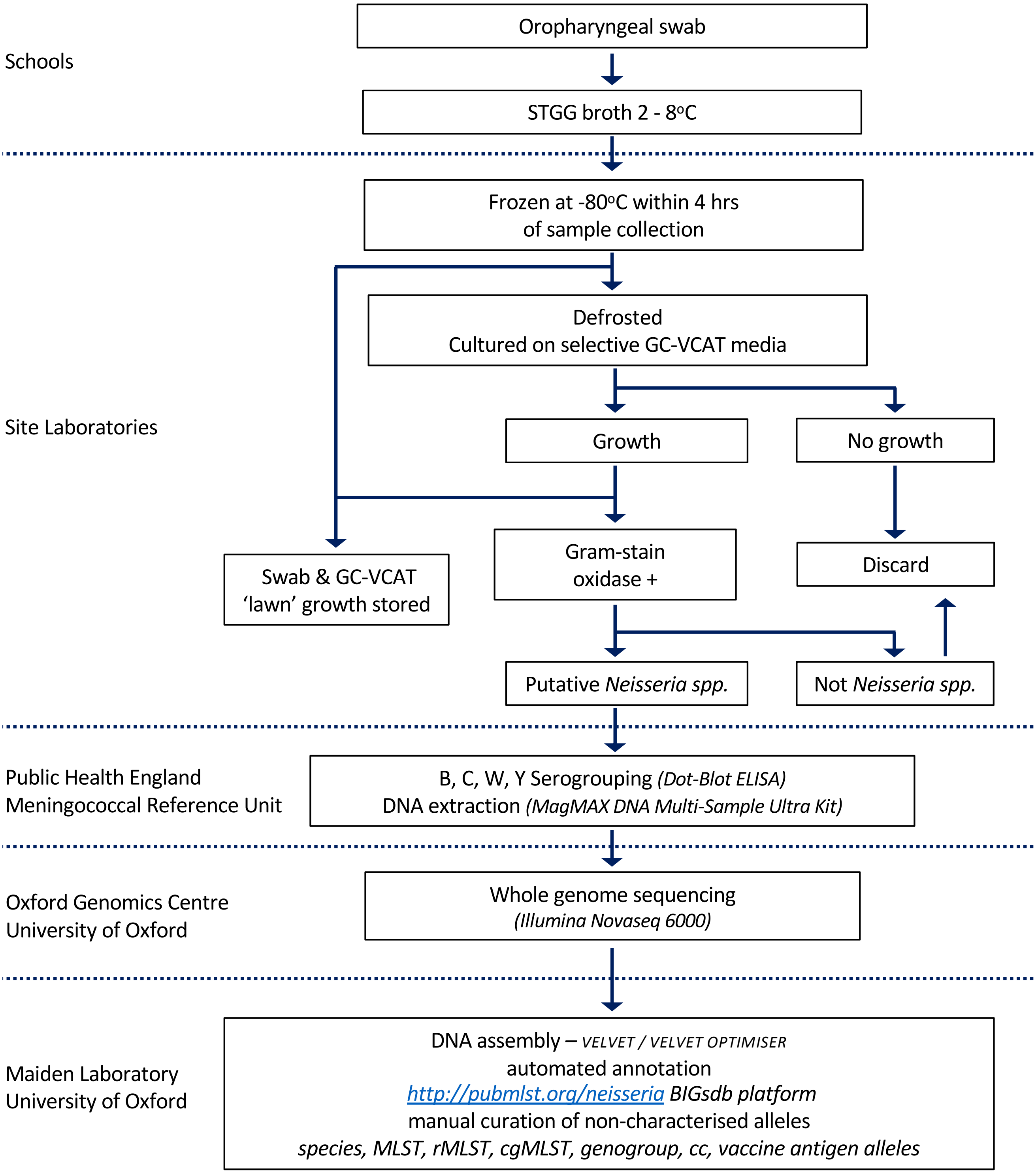
Schools
Oropharyngeal swab
STGG broth 2 - 8ºC
Site Laboratories
Frozen at -80ºC within 4 hrs
of sample collection
Defrosted
Cultured on selective GC-VCAT media
Growth
No growth
Swab & GC-VCAT
'lawn' growth stored
Gram-stain
oxidase +
Discard
Putative Neisseria spp.
Not Neisseria spp.
Public Health England
Meningococcal Reference Unit
B, C, W, Y Serogrouping (Dot-Blot ELISA)
DNA extraction (MagMAX DNA Multi-Sample Ultra Kit)
Oxford Genomics Centre
University of Oxford
Whole genome sequencing
(Illumina Novaseq 6000)
Maiden Laboratory
University of Oxford
DNA assembly – VELVET / VELVET OPTIMISER
automated annotation
http://pubmlst.org/neisseria BIGsdb platform
manual curation of non-characterised alleles
species, MLST, rMLST, cgMLST, genogroup, cc, vaccine antigen alleles

# Be on the TEAM: Teenagers against Meningitis

## Evaluating the effect of immunisation with group B meningococcal vaccines on meningococcal carriage

**Participant Number:** |__| |__| |__| |__| |__|

*****This information is confidential. It will not be seen by school staff or other students *****

**Q1. What is your current age in years?** 16 ❑ 17 ❑ 18 ❑ 19 ❑

**Q2. Are you:** male ❑ female ❑ non-binary ❑

**Q3. What is your ethnic group?**

White ❑
Asian/Asian British ❑
Black/African/Caribbean/Black British ❑
Mixed/multiple ethnic ❑
Other ethnic group ❑

**Q4. Do you currently have a cold or sore throat?** Yes ❑ No ❑

**Q5. Are you currently taking or have you recently stopped taking antibiotics?**

Not taken in the past month ❑
Stopped in the last month ❑
Stopped in the last week ❑
Yes currently taking ❑

**Q6. How many cigarettes do you smoke in a typical day?**

0 ❑ 1-5 ❑ 6-10 ❑ 11-20 ❑ more than 20 ❑

**Q7. Does any other person at home smoke cigarettes?** No ❑ Yes inside the house ❑ Yes outside the house ❑

**Q8. Do you vape (use e-cigarettes)?** Yes ❑ No ❑
**If YES: How many 10ml refills would you use in a week?** <1 ❑ 1-2 ❑ 3-4 ❑ 5-6 ❑ more than 7 ❑
**What percentage nicotine do you use most commonly?** 0-3 ❑ 4-7 ❑ 8-11 ❑ more than 12 ❑ don't know ❑

**Q9. How many days in the last week have you been to a party, pub, bar or nightclub?**

0 ❑ 1 ❑ 2-3 ❑ 4-5 ❑ 6-7 ❑

**Q10. How many people have you kissed (kissing with tongues, not just lips or cheeks) in the last week?**

0 ❑ 1 ❑ 2-3 ❑ 4 or more ❑

**Q11. Do you have a regular boyfriend or girlfriend?** Yes❑ No❑

**If YES: Do they smoke cigarettes?** Yes ❑ No ❑ don't know ❑

**Or do they vape (use e-cigarettes)?** Yes ❑ No ❑ don't know ❑

**Q12. Have you ever had the Men ACWY vaccine, also known as Nimenrix or Menveo (when you were 13 or 14 years old)?** No ❑ Yes ❑ Unknown ❑

**Thank you for completing this questionnaire**

For peer review only - http://bmjopen.bmj.com/site/about/guidelines.xhtml

# Reporting checklist for protocol of a clinical trial.

Based on the SPIRIT guidelines.

## Instructions to authors

Complete this checklist by entering the page numbers from your manuscript where readers will find each of the items listed below.

Your article may not currently address all the items on the checklist. Please modify your text to include the missing information. If you are certain that an item does not apply, please write "n/a" and provide a short explanation.

Upload your completed checklist as an extra file when you submit to a journal.

In your methods section, say that you used the SPIRITreporting guidelines, and cite them as:

Chan A-W, Tetzlaff JM, Altman DG, Laupacis A, Gøtzsche PC, Krleža-Jerić K, Hróbjartsson A, Mann H, Dickersin K, Berlin J, Doré C, Parulekar W, Summerskill W, Groves T, Schulz K, Sox H, Rockhold FW, Rennie D, Moher D. SPIRIT 2013 Statement: Defining standard protocol items for clinical trials. Ann Intern Med. 2013;158(3):200-207

| | | Reporting Item | Page Number |
|---|---|---|---|
| **Administrative information** | | | |
| Title | #1 | Descriptive title identifying the study design, population, interventions, and, if applicable, trial acronym | 1 |
| Trial registration | #2a | Trial identifier and registry name. If not yet registered, name of intended registry | 7 |
| Trial registration: data set | #2b | All items from the World Health Organization Trial Registration Data Set | n/a |
| Protocol version | #3 | Date and version identifier | 25 |
| Funding | #4 | Sources and types of financial, material, and other support | 24 |
| Roles and | #5a | Names, affiliations, and roles of protocol | 24 |

For peer review only

| | | | |
|---|---|---|---|
| responsibilities: contributorship | | contributors | |
| Roles and responsibilities: sponsor contact information | #5b | Name and contact information for the trial sponsor | 20 |
| Roles and responsibilities: sponsor and funder | #5c | Role of study sponsor and funders, if any, in study design; collection, management, analysis, and interpretation of data; writing of the report; and the decision to submit the report for publication, including whether they will have ultimate authority over any of these activities | 24 |
| Roles and responsibilities: committees | #5d | Composition, roles, and responsibilities of the coordinating centre, steering committee, endpoint adjudication committee, data management team, and other individuals or groups overseeing the trial, if applicable (see Item 21a for data monitoring committee) | 20,23 |
| **Introduction** | | | |
| Background and rationale | #6a | Description of research question and justification for undertaking the trial, including summary of relevant studies (published and unpublished) examining benefits and harms for each intervention | 9-12 |
| Background and rationale: choice of comparators | #6b | Explanation for choice of comparators | 12 |
| Objectives | #7 | Specific objectives or hypotheses | 12-13 |
| Trial design | #8 | Description of trial design including type of trial (eg, parallel group, crossover, factorial, single group), allocation ratio, and framework (eg, superiority, equivalence, non-inferiority, exploratory) | 12-13,16 |
| **Methods: Participants,** | | | |

For peer review only

| | | | |
|---|---|---|---|
| **interventions, and outcomes** | | | |
| Study setting | #9 | Description of study settings (eg, community clinic, academic hospital) and list of countries where data will be collected. Reference to where list of study sites can be obtained | 13 |
| Eligibility criteria | #10 | Inclusion and exclusion criteria for participants. If applicable, eligibility criteria for study centres and individuals who will perform the interventions (eg, surgeons, psychotherapists) | 14 |
| Interventions: description | #11a | Interventions for each group with sufficient detail to allow replication, including how and when they will be administered | 17 |
| Interventions: modifications | #11b | Criteria for discontinuing or modifying allocated interventions for a given trial participant (eg, drug dose change in response to harms, participant request, or improving / worsening disease) | 17, 20 |
| Interventions: adherance | #11c | Strategies to improve adherence to intervention protocols, and any procedures for monitoring adherence (eg, drug tablet return; laboratory tests) | 19 |
| Interventions: concomitant care | #11d | Relevant concomitant care and interventions that are permitted or prohibited during the trial | n/a |
| Outcomes | #12 | Primary, secondary, and other outcomes, including the specific measurement variable (eg, systolic blood pressure), analysis metric (eg, change from baseline, final value, time to event), method of aggregation (eg, median, proportion), and time point for each outcome. Explanation of the clinical relevance of chosen efficacy and harm outcomes is strongly recommended | 12, 13 |
| Participant timeline | #13 | Time schedule of enrolment, interventions (including any run-ins and washouts), assessments, and visits for participants. A schematic diagram is highly recommended (see Figure) | 14 |

For peer review only

| | | | |
|---|---|---|---|
| Sample size | #14 | Estimated number of participants needed to achieve study objectives and how it was determined, including clinical and statistical assumptions supporting any sample size calculations | 13 |
| Recruitment | #15 | Strategies for achieving adequate participant enrolment to reach target sample size | 13,14 |
| **Methods: Assignment of interventions (for controlled trials)** | | | |
| Allocation: sequence generation | #16a | Method of generating the allocation sequence (eg, computer-generated random numbers), and list of any factors for stratification. To reduce predictability of a random sequence, details of any planned restriction (eg, blocking) should be provided in a separate document that is unavailable to those who enrol participants or assign interventions | 16 |
| Allocation concealment mechanism | #16b | Mechanism of implementing the allocation sequence (eg, central telephone; sequentially numbered, opaque, sealed envelopes), describing any steps to conceal the sequence until interventions are assigned | n/a |
| Allocation: implementation | #16c | Who will generate the allocation sequence, who will enrol participants, and who will assign participants to interventions | 16 |
| Blinding (masking) | #17a | Who will be blinded after assignment to interventions (eg, trial participants, care providers, outcome assessors, data analysts), and how | n/a |
| Blinding (masking): emergency unblinding | #17b | If blinded, circumstances under which unblinding is permissible, and procedure for revealing a participant's allocated intervention during the trial | n/a |
| **Methods: Data collection,** | | | |

For peer review only

| | | | |
|---|---|---|---|
| **management, and analysis** | | | |
| Data collection plan | #18a | Plans for assessment and collection of outcome, baseline, and other trial data, including any related processes to promote data quality (eg, duplicate measurements, training of assessors) and a description of study instruments (eg, questionnaires, laboratory tests) along with their reliability and validity, if known. Reference to where data collection forms can be found, if not in the protocol | 16-17, 21 |
| Data collection plan: retention | #18b | Plans to promote participant retention and complete follow-up, including list of any outcome data to be collected for participants who discontinue or deviate from intervention protocols | 21 |
| Data management | #19 | Plans for data entry, coding, security, and storage, including any related processes to promote data quality (eg, double data entry; range checks for data values). Reference to where details of data management procedures can be found, if not in the protocol | 21 |
| Statistics: outcomes | #20a | Statistical methods for analysing primary and secondary outcomes. Reference to where other details of the statistical analysis plan can be found, if not in the protocol | 20-21 |
| Statistics: additional analyses | #20b | Methods for any additional analyses (eg, subgroup and adjusted analyses) | 20-21 |
| Statistics: analysis population and missing data | #20c | Definition of analysis population relating to protocol non-adherence (eg, as randomised analysis), and any statistical methods to handle missing data (eg, multiple imputation) | 20-21 |
| **Methods: Monitoring** | | | |
| Data monitoring: formal committee | #21a | Composition of data monitoring committee (DMC); summary of its role and reporting structure; | 20. |

For peer review only

| | | | |
|---|---|---|---|
| | | statement of whether it is independent from the sponsor and competing interests; and reference to where further details about its charter can be found, if not in the protocol. Alternatively, an explanation of why a DMC is not needed | |
| Data monitoring: interim analysis | #21b | Description of any interim analyses and stopping guidelines, including who will have access to these interim results and make the final decision to terminate the trial | 20-21 |
| Harms | #22 | Plans for collecting, assessing, reporting, and managing solicited and spontaneously reported adverse events and other unintended effects of trial interventions or trial conduct | 20 |
| Auditing | #23 | Frequency and procedures for auditing trial conduct, if any, and whether the process will be independent from investigators and the sponsor | 20 & full protocol |
| **Ethics and dissemination** | | | |
| Research ethics approval | #24 | Plans for seeking research ethics committee / institutional review board (REC / IRB) approval | 25 |
| Protocol amendments | #25 | Plans for communicating important protocol modifications (eg, changes to eligibility criteria, outcomes, analyses) to relevant parties (eg, investigators, REC / IRBs, trial participants, trial registries, journals, regulators) | 25 |
| Consent or assent | #26a | Who will obtain informed consent or assent from potential trial participants or authorised surrogates, and how (see Item 32) | 15 & 16 |
| Consent or assent: ancillary studies | #26b | Additional consent provisions for collection and use of participant data and biological specimens in ancillary studies, if applicable | 15 & 16 |
| Confidentiality | #27 | How personal information about potential and enrolled participants will be collected, shared, and | 21 |

For peer review only

| | | | |
|---|---|---|---|
| | | maintained in order to protect confidentiality before, during, and after the trial | |
| Declaration of interests | #28 | Financial and other competing interests for principal investigators for the overall trial and each study site | 25 |
| Data access | #29 | Statement of who will have access to the final trial dataset, and disclosure of contractual agreements that limit such access for investigators | 26 |
| Ancillary and post trial care | #30 | Provisions, if any, for ancillary and post-trial care, and for compensation to those who suffer harm from trial participation | n/a detailed in full protocol & patient information sheet |
| Dissemination policy: trial results | #31a | Plans for investigators and sponsor to communicate trial results to participants, healthcare professionals, the public, and other relevant groups (eg, via publication, reporting in results databases, or other data sharing arrangements), including any publication restrictions | 26 |
| Dissemination policy: authorship | #31b | Authorship eligibility guidelines and any intended use of professional writers | 26 |
| Dissemination policy: reproducible research | #31c | Plans, if any, for granting public access to the full protocol, participant-level dataset, and statistical code | 26 |
| **Appendices** | | | |
| Informed consent materials | #32 | Model consent form and other related documentation given to participants and authorised surrogates | 26 & study website |
| Biological specimens | #33 | Plans for collection, laboratory evaluation, and storage of biological specimens for genetic or molecular analysis in the current trial and for future use in ancillary studies, if applicable | 18-19 |

Notes:

For peer review only

- 30: n/a detailed in full protocol & patient information sheet
- 32: 26 & study website The SPIRIT checklist is distributed under the terms of the Creative Commons Attribution License CC-BY-ND 3.0. This checklist was completed on 30. January 2020 using https://www.goodreports.org/, a tool made by the EQUATOR Network in collaboration with Penelope.ai

For peer review only
